# Self-Assembly of Covalently Linked Porphyrin Dimers at the Solid–Liquid Interface

**DOI:** 10.3390/molecules24163018

**Published:** 2019-08-20

**Authors:** Thomas Habets, Dennis Lensen, Sylvia Speller, Johannes A.A.W. Elemans

**Affiliations:** 1 Institute for Molecules and Materials, Radboud University, Heyendaalseweg 135, 6525 AJ Nijmegen, The Netherlands; 2 Institute of Physics, University of Rostock, Albert-Einstein-Straße 23, 18059 Rostock, Germany; 3 Department Life, Light & Matter—University of Rostock, 18051 Rostock, Germany

**Keywords:** porphyrins, self-assembly, scanning tunneling microscopy, surfaces

## Abstract

The synthesis and surface self-assembly behavior of two types of metal-porphyrin dimers is described. The first dimer type consists of two porphyrins linked via a rigid conjugated spacer, and the second type has an alkyne linker, which allows rotation of the porphyrin moieties with respect to each other. The conjugated dimers were equipped with two copper or two manganese centers, while the flexible dimers allowed a modular built-up that also made the incorporation of two different metal centers possible. The self-assembly of the new porphyrin dimers at a solid–liquid interface was investigated at the single-molecule scale using scanning tunneling microscopy (STM). All dimers formed monolayers, of which the stability and the internal degree of ordering of the molecules depended on the metal centers in the porphyrins. While in all monolayers the dimers were oriented coplanar with respect to the underlying surface (‘face-on’), the flexible dimer containing a manganese and a copper center could be induced, via the application of a voltage pulse in the STM setup, to self-assemble into monolayers in which the porphyrin dimers adopted a non-common perpendicular (‘edge-on’) geometry with respect to the surface.

## 1. Introduction

Metal-porphyrins occur ubiquitously in nature, e.g., as magnesium porphyrin pigments in the light harvesting system [1,2] and as iron porphyrins in the blood protein hemoglobin [3] and the oxidation enzyme cytochrome P450 [4,5]. In biomimetic chemistry, many artificial porphyrin systems have been developed with the aim to approach the efficient functional properties of their natural counterparts, for instance in areas of molecular electronics [6,7,8] and catalysis [9,10,11]. In the past decades, our group has extensively investigated the catalytic properties of Mn(III) porphyrins in the epoxidation of alkenes [12,13,14,15,16]. The majority of these studies have been carried out using supramolecular systems in solution, and in order to obtain more detailed information about the reaction mechanisms during catalysis, we have also investigated the behavior of single porphyrin molecules adsorbed at a surface. To this end, we constructed self-assembled monolayers of metal-porphyrins at a solid–liquid interface and investigated their properties with scanning tunneling microscopy (STM) [17,18,19,20]. This technique does not only enable the probing of structural properties of single molecules but also provides electronic information, e.g., about changes in the redox state of the porphyrin metal center during a chemical reaction. Furthermore, a solid–liquid interface environment closely resembles the reaction conditions encountered in laboratory-scale chemical reactions: Room temperature, atmospheric pressure, and the presence of a liquid to which reagents can easily be added and products withdrawn. For these reasons, STM at a solid–liquid interface has evolved as a tool to analyze chemical reactions at single-molecule scale, which is complementary to conventional ensemble techniques such as NMR spectroscopy and mass spectrometry [21].

In previous work, we had constructed a variety of supramolecular assemblies of (metal-) porphyrins at solid–liquid interfaces and imaged their spatial organization with the help of liquid-STM [7]. By applying specific molecular design, we were able to precisely control the two-dimensional (2D) polymorphic behavior of these assemblies by varying the nature or the concentration of the solution in the STM experiments [22,23,24]. We also constructed 2D assemblies of catalytically active Mn(III) porphyrins and investigated their reactivity with oxygen and the epoxidation of alkenes at the single-molecule scale [25,26]. While these porphyrins are inert in solution, their adsorption to a negatively biased surface in the STM setup (or the application of a voltage pulse [27]) induced the reduction of the metal centers from Mn(III) to Mn(II), which was accompanied by a topographic signature change of the molecules in the STM images and which resulted in the emergence of reactivity towards molecular oxygen (O_2_). Interestingly, STM revealed that O_2_ reacted in a cooperative fashion with the manganese porphyrins, in the sense that the adsorbed catalysts were able to coordinate O_2_ to the metal center, dissociate the oxygen-oxygen bond, and subsequently distribute the two oxygen atoms over two adjacent metal-porphyrins at the surface, generating Mn(IV)-oxo complexes [25,26]. These complexes appeared in the STM images as signatures with a significantly increased apparent height compared to the signatures attributed to the Mn(III) and Mn(II) porphyrins, highlighting the ability of the technique to map both structural and electronic changes during chemical reactions.

**Figure 1:**
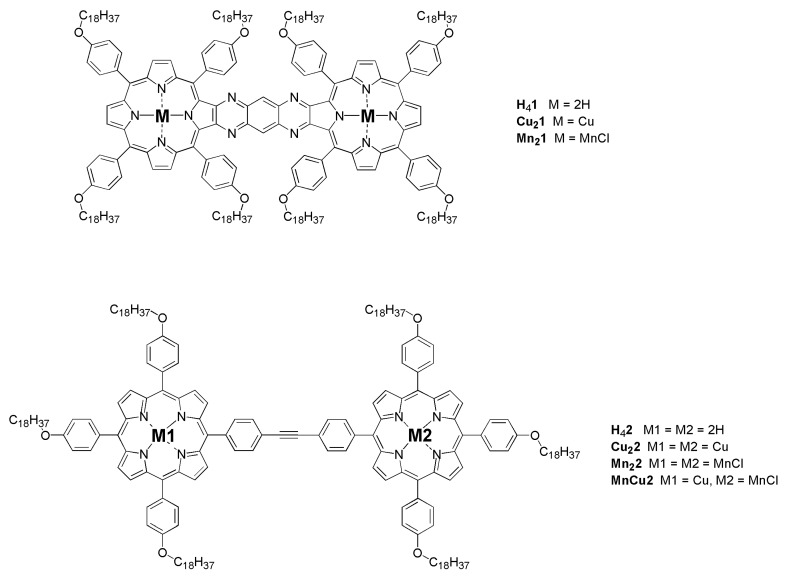
Molecular structures of porphyrin dimers with a rigid conjugated linker (top) and with a freely rotatable alkyne linker (bottom).

Intrigued by the cooperative reactivity of manganese porphyrins adsorbed at adjacent locations at the solid–liquid interface, we decided to design and synthesize covalently connected metal-porphyrins, with the ultimate aim to investigate with STM the possible cooperativity between two metal centers in the same molecule. Several examples of covalently linked porphyrin dimers have been described [28,29,30,31,32] and some have been imaged with STM in an ultra-high vacuum environment or at a solid–liquid interface [33,34,35,36,37]. Our intention to adsorb porphyrin dimers as immobilized assemblies at a solid–liquid interface required the attachment of long aliphatic chains, which are known to aid assembly as a result of van der Waals interactions with the surface and intermolecular alkane chain interdigitation [38]. We have designed two types of porphyrin dimers (Figure 1), either connected via a rigid conjugated system (compounds **1**) or via a relatively flexible alkyne linker (compounds **2**). We had inserted Mn(III) centers into the dimers because of their potential redox and catalytic properties, and Cu(II) centers that did not show redox or catalytic behavior and may thus serve as a reference. It should be noted that, unlike copper porphyrins, manganese porphyrins carry a counter anion, which in our case is an inner shell axial chloride. In a previous communication, we had already reported the synthesis of flexible dimers **H_4_2**, **Cu2,** and **MnCu2** [39]. While STM revealed that **Cu2** formed highly ordered monolayers at a solid–liquid interface, **MnCu2** turned out to be able to self-assemble into stacked bilayers in which the porphyrin dimers in the two layers were ordered in a specific geometry with respect to each other. In this paper, we present the synthesis of **Mn_2_2** and of the rigid conjugated dimers **H_4_1**, **Cu_2_1,** and **Mn_2_1**, and STM studies of the self-assembly behavior of the metal-porphyrin dimers at the solid–liquid interface. We are particularly interested in the effect of the presence of the axial chloride ligands at the manganese porphyrin dimers on the ability of the dimers to form stable adlayers, since these ions may inhibit favorable cofacial adsorption of the porphyrin planes with respect to the underlying surface. Finally, we report an unexpected change in two-dimensional (2D) surface polymorphism of **MnCu2** upon the application of mild voltage pulses to the solid–liquid interface, which caused the orientation of the porphyrin planes to switch from a ‘face-on’ to an ‘edge-on’ geometry with respect to the underlying graphite surface.

## 2. Results and Discussion

### 2.1. Synthesis

**Scheme 1:**
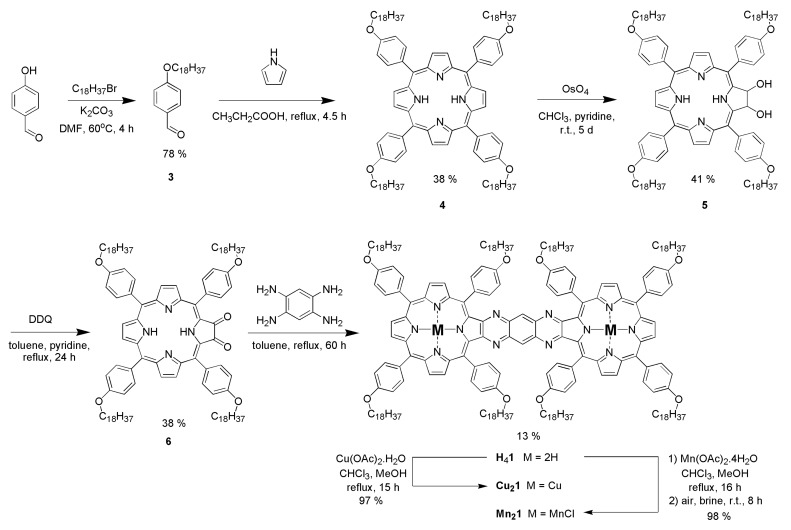
Synthesis of porphyrin dimers with a rigid conjugated linker, and their metal derivatives.

Conjugated porphyrin dimer **H_4_1** was synthesized in five steps, starting from 4-hydroxybenzaldehyde, which was alkylated with 1-bromooctadecane to give **3** in 78% yield (Scheme 1). This compound was subsequently condensed with pyrrole to give porphyrin **4** in 38% yield. Using a modified previously reported protocol [40], **4** was regioselectively dihydroxylated at one of its β-pyrrole moieties using osmium tetraoxide to give diol **5** in 41% yield. This compound was oxidized with 2,3-dichloro-5,6-dicyano-1,4-benzoquinone (DDQ) to diketone **6** in 38% yield. Finally, two equivalents of **6** were condensed with one equivalent of 1,2,4,5-tetraaminobenzene to give the desired bis-porphyrin **H_4_1** in 38% yield. Compound **H_4_1** could be readily converted into **Cu_2_1** by reacting it with copper(II) acetate, and into **Mn_2_2** by reacting it with manganese(II) acetate tetrahydrate followed by aerobic oxidation in brine (both in nearly quantitative yields). The synthesis of acetylene-linked porphyrin dimer **H_4_2** and its metal complexes **Cu_2_2** and **MnCu2** has been reported by us previously [39]. Bis-manganese dimer **Mn_2_2** was synthesized in nearly quantitative yield from **H_4_2** following the same procedure as for the synthesis of **Mn_2_1**. The progress of all-metal insertion reactions was monitored by UV-vis spectroscopy, in which chloroform solutions of the reaction mixtures were acidified with concentrated aqueous HCl; as soon as absorptions correlated to protonated free base porphyrins remained absent, the metallation was considered complete and the reaction stopped (see Supplementary Materials for spectral data of all new compounds).

### 2.2. Self-Assembly of Conjugated Porphyrin Dimers **Cu_2_1** and **Mn_2_1** at a Solid–Liquid Interface

**Figure 2:**
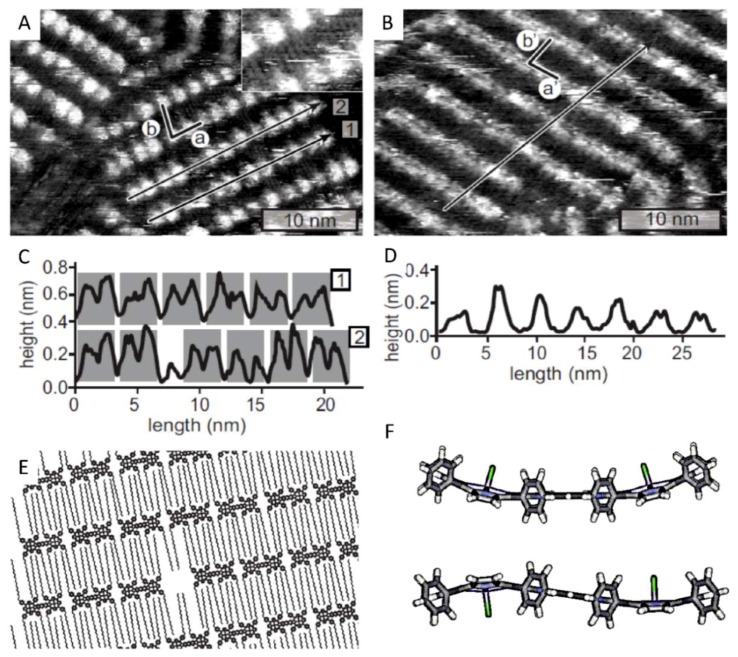
(**A**) Scanning tunneling microscopy (STM) image of a self-assembled monolayer of **Cu_2_1** at the highly oriented pyrolytic graphite (HOPG)/1-octanoic acid interface. The unit cell is indicated by vectors **a** and **b**. The inset shows a magnification. The black lines represent two cross-sections which are shown in panel (**C**). Tunneling parameters: V_bias_ = −350 mV, I_t_ = 5.5 pA. (**B**) STM image of a self-assembled monolayer of **Mn_2_1** at the HOPG/1-octanoic acid interface. The unit cell is indicated by vectors **a’** and **b’**. The black line represents the cross-section shown in panel (**D**). Tunneling parameters: V_bias_ = −450 mV, I_t_ = 3.2 pA. (**E**) Proposed molecular model of the organization of both porphyrin dimers at the interface. (**F**) Molecular models of the aromatic framework of **Mn_2_1** (side view) in which the two axial chloride ligands are either oriented syn (top) or anti (bottom) with respect to each other.

In Figure 2, STM images of self-assembled monolayers of **Cu_2_1** and **Mn_2_1** at the highly oriented pyrolytic graphite (HOPG)/1-octanoic acid interface are shown. Droplets of 3–5 μL with concentrations of ~10^−5^ M of the compounds in 1-octanoic acid were applied to a freshly cleaved HOPG surface. Only in approximately 20% of the attempts, monolayers could be successfully imaged at (sub)molecular resolution. In all other cases, typically poorly resolved structures were found revealing at best the presence of vague lamellar arrays. This is probably due to incomplete assembly of the molecules in a fully packed layer, or to fast adsorption/desorption kinetics [41,42,43] of the molecules at the solid–liquid interface, preventing the formation of a layer that is immobilized enough to be resolvable by STM.

The orientation of the dimers of **Cu_2_1** within the self-assembled monolayer can be derived from the image in Figure 2A. Based on their shape and size, and taking into account the arrangement of the side-groups, each of the bright-appearing (i.e., elevated) spots is assigned to a porphyrin moiety that is oriented with its aromatic plane in a largely coplanar geometry with respect to the surface. Consequently, two of such signatures may constitute one porphyrin dimer molecule. The dimers are aligned with their long axis along the lamellae, as is shown in the proposed structural model in Figure 2E. This organization was concluded by considering the distances between the porphyrin moieties along and perpendicular to the lamella, respectively, especially the observation of alternating small and large spacings. The unit cell of the monolayer (drawn in Figure 2A) is defined by vectors **a** = 3.5 ± 0.2 and **b** = 3.9 ± 0.2 nm, with an angle of 79 ± 8° between them. Based on the distance between the lamellae, the C_18_-alkyl chains of adjacent porphyrin dimers have to be interdigitated. In some parts of the image, these chains are discernable in the darker regions between the bright signatures (inset in Figure 2A), and careful analysis revealed that they are fully adsorbed on the HOPG surface in an interdigitated geometry. They are, however, not always ordered alternately, i.e., one chain originating from a dimer in a lamellar array is not necessarily followed on the surface by a chain of a dimer from an adjacent lamella. This irregular distribution is also shown in the model in Figure 2E. The chains are organized in pairs as a result of the limited spacing between the porphyrin moieties within one dimer, which does not allow interdigitation of a chain from a dimer in an adjacent lamella. Based on the orientation of the dimers, one would expect to observe an even number of bright signatures along each lamella within a monolayer domain. Overall, this is indeed observed in the monolayer of **Cu_2_1**, for example when the lamella along line 1 in Figure 2A is considered. However, the lamella along line 2 reveals an odd number of bright signatures. The cross-sections of both lines are shown in Figure 2C. Cross-section 1 shows the apparent height profile of six dimers, which show up as twelve protrusions, each corresponding to a porphyrin moiety within the dimers. The gray boxes indicate the separate dimers. The cross-section furthermore shows that the depression in apparent height between the porphyrin moieties in a dimer is less deep than the depression between the porphyrin moieties of two adjacent dimers, which is the basis of our assignment. If we now consider cross-section 2, dimers can also be assigned based on the apparent height of the porphyrin moieties. Again the gray boxes indicate which of the protrusions form a dimer. Based on this analysis, the 5th protrusion from the left, with a width of about 1.8 nm, is not a fragment of a dimer. We, therefore, assign this feature to a vacant site, although the small protrusion may be caused by one or more trapped and poorly resolved solvent molecules [44,45]. The model in Figure 2E also shows such a vacancy. Since the alkyl chains are not ordered in an alternating fashion, the next bright signature available is at a distance half a dimer distance further, at ½**a** = 1.75 ± 0.1 nm, which is in agreement with the width of the described vacancy feature.

Figure 2B shows an STM image of a self-assembled monolayer of **Mn_2_1** at the HOPG/1-octanoic acid interface. Although the resolution is not as good as that of the monolayer of **Cu_2_1**, it is clear that also **Mn_2_1** self-assembles into a lamellar structure, with unit cell vectors **a′** = 3.4 ± 0.4 and **b′** = 3.9 ± 0.3 nm, under an angle of 80° ± 10°. Within experimental error, these values are the same as those of the unit cell of **Cu_2_1**, indicating that the substitution of the metal centers does not change the spatial ordering of the dimers on the HOPG surface. The difference in resolution between the monolayers of **Cu_2_1** and **Mn_2_1** may be explained by the presence of the axial chloride ligand at the manganese centers of **Mn_2_1**. This ligand may hinder the adsorption of the dimers at the surface, or alter the tunneling pathway between the surface and the tip. The self-assembled monolayers of **Mn_2_1** appeared to be not very stable. They only persisted when the tunnel current was maintained below 10 pA, and even then they typically disappeared after about 30 min of scanning. Although the resolution is not optimal, the signatures of some of the manganese porphyrin moieties appear to differ in brightness. The cross-section in Figure 2D shows an apparent height difference of 0.1 nm between bright and less bright appearing porphyrins. The brighter signatures have a radius of 1.2 nm, and thus span only one manganese porphyrin, not the whole dimer structure. Due to the lack of sub-molecular resolution, it is not possible to determine whether more than two different signatures are present in the monolayer. So far, the origin of the different signatures is unclear. They might be the result of redox reactions occurring at the manganese centers. Unlike the Cu(II) centers of **Cu_2_1**, the Mn(III) centers of **Mn_2_1** may undergo reduction to Mn(II), or oxidation to Mn(IV) upon reaction with oxygen, as has been demonstrated in our previous STM work on monomeric manganese porphyrins [25,26]. Such reactions would most certainly lead to a difference in the appearance of the porphyrins in the STM images. An alternative explanation would be that the two porphyrins within an **Mn_2_1** dimer adsorb in a different geometry to the underlying surface. Since the manganese centers have an axially coordinated chloride ligand, **Mn_2_1** can exist in two isomeric forms, at least in principle. Assuming kinetically stable coordination of the inner shell chloride ligand to the metal center, a molecule of **Mn_2_1** can have both chloride ligands coordinated at the same (syn-isomer) or at the opposite face (anti-isomer) of a porphyrin dimer (Figure 2F). Due to the paramagnetic nature of the manganese centers, the existence or abundance of these isomers could not be investigated with the help of NMR spectroscopy. The rigid, conjugated linker between the porphyrin moieties furthermore inhibits isomerization, via rotation, from the syn- to the anti-isomer and vice versa. Assuming the existence of both isomers, one can envisage that they will adsorb in different geometries at the solid–liquid interface. While the conjugated porphyrin plane of the syn-isomer can adsorb in a cofacial geometry with respect to the surface, this is impossible for the anti-isomer since one axial chloride ligand will always inhibit this geometry. In the latter case, the dimer may be adsorbed in a geometry in which it is lifted on the side where the chloride ligand points in the direction of the surface. As a result of this tilted geometry, the amount of π−π stacking and van der Waals interactions between the surface and the molecules is not ideal, which may be an explanation for the limited stability of the monolayer structure in time.

### 2.3. Self-Assembly of the Non-Conjugated Porphyrin Dimers **Cu_2_2**, **Mn_2_2**, and **MnCu2** at a Solid–Liquid Interface

In a previous paper [39] we reported the self-assembly properties of **Cu_2_2** and **MnCu2** at the solid–liquid interface. Both compounds formed stable and highly organized monolayers, and in some cases, bilayer formation was observed for **MnCu2**, in which the dimers were stacked in two 2D layers on top of each other. The top 2D layer was found to be able to laterally move stepwise with respect to the bottom 2D layer, which was strongly immobilized to the HOPG surface. The fact that the molecules of **MnCu2** formed stable and extended uniform 2D layers of molecules that were oriented with their porphyrin planes coplanar with the underlying HOPG substrate, indicates that the single axial chloride ligand at the manganese centers of these molecules does not hinder such an adsorption geometry. It was therefore of interest to investigate the assembly behavior of **Mn_2_2**, which has two of these chloride ligands. 

**Figure 3:**
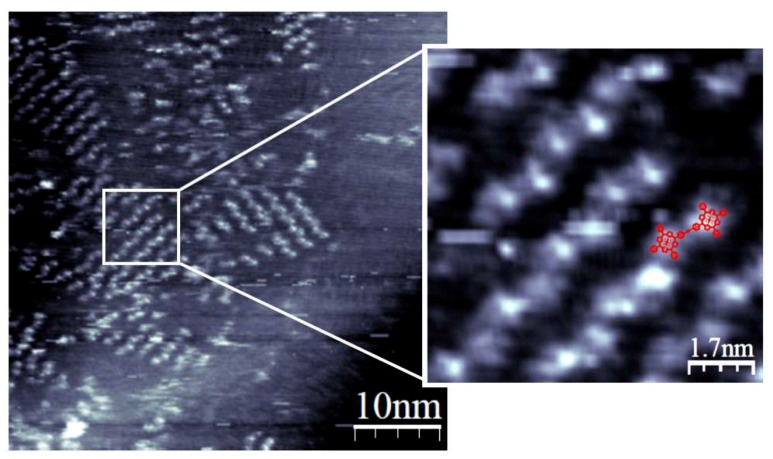
STM image and magnification of a self-assembled monolayer of **Mn_2_2** at the HOPG/1-octanoic acid interface. A schematic dimer of **Mn_2_2** is superimposed in red in the magnification. Tunneling parameters: V_bias_ = −500 mV, I_t_ = 2.5 pA.

It turned out to be very difficult to obtain assemblies of **Mn_2_2** at the HOPG/1-octanoic acid interface that were stable enough to be imaged with STM. In most cases, only vague and very dynamic structures were observed and no reliable unit cell could be determined. Only sparingly, small patches (<30 × 30 nm^2^) emerged, which were not as close-packed as those of **Cu_2_2** or **MnCu2** (Figure 3). The layers were even more unstable than those of the rigid conjugated dimer **Mn_2_1** and typically disappeared within several minutes. Like **Mn_2_1**, also **Mn_2_2** can exist as isomers in which the two chloride ligands are oriented in different directions with respect to each other. However, unlike in the case of **Mn_2_1**, the isomers of **Mn_2_2** can interconvert since the two porphyrin moieties can freely rotate with respect to each other about the alkyne linker. The fact that **MnCu2** forms extended and stable assemblies and **Mn_2_2** does not, and the fact that also layers of **Mn_2_1** are unstable, suggests that the presence of two axial chloride ligands within a given manganese porphyrin dimer inhibits the formation of stable layers at a surface. This may be the result of a higher stability of dimers with an anti-configuration of the two ligands compared to those with a syn-configuration, which in turn may be governed by a lower dipole moment in the former isomers. 

### 2.4. 2D Polymorphism of Porphyrin Dimer **MnCu2**

**Figure 4:**
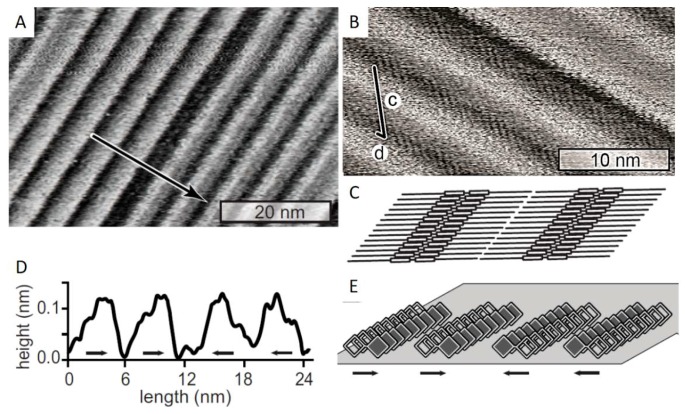
(**A**) and (**B**): STM images of a monolayer of **MnCu2** at the HOPG/1-phenyloctane interface; the unit cell vectors **c** and **d** are drawn in panel (**B**); tunneling parameters: (**A**) V_bias_ = −550 mV, I_t_ = 50 pA, (**B**) V_bias_ = 450 mV, I_t_ = 50 pA. (**C**) Schematic representation (top view) of the proposed ‘edge-on’ orientation of the molecules of **MnCu2** on HOPG. (**D**) Cross-section corresponding to the black line drawn in panel (**A**); the small black arrows point in the direction of the brighter parts of the lamellae. (**E**) Schematic representation of one of the proposed orientations of the molecules of **MnCu2**; the filling of the squares represent the different metal porphyrin moieties and the small black arrows a possible directionality within the stacks.

During the STM measurements of porphyrin dimer **MnCu2**, we observed an interesting case of 2D polymorphism. In less than 10% of the attempts to image a self-assembled structure of this compound, no structures were observed at all at the HOPG surface. It is then common practice to induce monolayer formation by electric polarization, applying one or more mild voltage pulses to the tunnel junction, in this case between +1 and +2 V with a duration of 900 μs. In about 5% of these attempts, within 5 min a monolayer structure as shown in the STM images in Figure 4A–B grew over the whole scan area. After the other attempts, no structure emerged or the STM tip became too blunt to be used for further imaging. Although the STM images are inconclusive about the precise orientation of the dimers, it is plausible that they are oriented with their porphyrin planes perpendicular (‘edge-on’) to the surface, as is drawn in the schematic representation in Figure 4C, in which each rectangle corresponds to one porphyrin moiety within a dimer. 2D polymorphic changes upon the application of a voltage pulse have been reported previously for other types of disk-shaped molecules adsorbed at a solid–liquid interface [46,47]. 

Similar as was observed in the other STM images of the porphyrin dimers, we propose that the bright lamellae correspond to the aromatic parts of **MnCu2** and the darker regions to the alkyl chains. Similar ‘edge-on’ orientation geometries at the solid–liquid interface have previously been observed for porphyrin oligomers containing six [17] and twelve porphyrin moieties [18,19], phthalocyanine derivatives [48], and other planar molecules with extended aromatic surfaces [49,50,51,52,53]. The formation of the ‘edge-on’ assemblies of **MnCu2** is most likely governed by stabilizing π-stacking between the aromatic porphyrin planes of the dimers. Despite the fact that individual dimers can be hardly discerned in Figure 4A–B, in the darker parts between the lamellae signatures are visible, which we propose to be the alkyl chains of the dimer molecules. Based on this assumption, a unit cell was determined with vectors **c** = 6.2 ± 0.3 and **d** = 0:53 ± 0.2 nm, under an angle of 48° ± 5°. These unit cell dimensions give no clue as to whether the alkyl chains of dimers in adjacent lamella are interdigitated or not. The unit cell parameters correspond to an orientation of the dimers as drawn in Figure 4C, i.e., in which the porphyrin rings are oriented in an off-set geometry, which is typical for π−π stacking of small molecules. From the parameters, a porphyrin stacking distance of about 4 Å can be calculated, which is a realistic value for stacked aromatic surfaces. Remarkably, the bright parts of the lamellae all show a brighter side and a less bright side, which can also be discerned in the cross-section drawn in Figure 4A,D. These differences in brightness may be explained by the presence of different metal centers in the two halves of the porphyrin dimers, and a unidirectional orientation within the lamellae. In the schematic drawing in Figure 4E, the squares representing the porphyrin moieties are shown either filled or empty, corresponding to porphyrin moieties with a manganese or a copper center, respectively. A unidirectional orientation would mean that in a given stack of porphyrin dimers the same metal centers are always aligned behind each other, resulting in an apparent height difference between the two halves of the lamella. Such an ordering might be governed by favorable dipole–dipole interactions between the Mn-Cl metal-ligand pairs of adjacent dimers. However, similar internal differences in lamellar brightness have previously been observed in STM images of porphyrin hexamers [17] and dodecamers [18,19] which contained no or identical metal centers. Another, and probably more likely explanation for the contrast differences in the lamellae of **MnCu2** may, therefore, be variations in the orientation of the stacks of the dimers with respect to the atomic ordering of the underlying solid surface. The ordering of the stacks may not be fully commensurate with the ordering of the underlying graphite, leading to the observed differences in the sub-lamellar signature.

The assemblies of ‘edge-on’-orientated dimers of **MnCu2** were exclusively observed after the application of a voltage pulse to the tunnel junction, which strongly indicates that the pulse plays a decisive role in their formation. A first explanation for this may be that the pulse removes the axial chloride ligands from the manganese porphyrin moieties, as has been proposed before for the dissociation of the same ligand from a single manganese porphyrin upon application of a voltage pulse to the interface of HOPG and a 1-octanoic acid solution of that compound [27]. Alternatively, or perhaps also concomitantly, the strong electric field involved with the pulse might align the dimers already in solution and induce the deposition a small seed of ‘edge-on’ oriented dimers at the solid–liquid interface. Subsequently, other porphyrin dimers can attach to this seed resulting in the growth of extended ‘edge-on’ patterns over the whole scanning area. The ‘edge-on’ patterns remained stable over time and no porphyrin dimers that were adsorbed coplanar (‘face-on’) to the surface were observed. 

**Figure 5:**
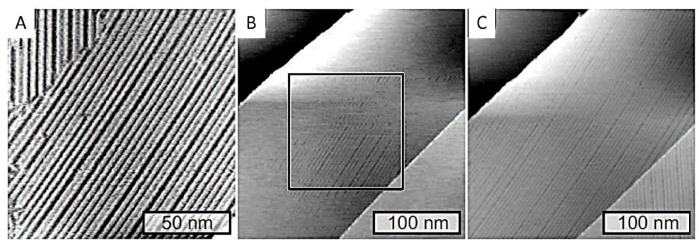
STM images showing the growth of an ‘edge-on’ domain of **MnCu2** at the HOPG/1-phenyloctane interface. (**A**) The pattern formed directly after the application of a voltage pulse. (**B**) Zoom-out, the black square corresponds with the scan area of the STM image in (**A**). (**C**) STM image of the same area as in (**B**) after 20 min of scanning. Tunneling parameters: V_bias_ = −450 mV, I_t_ = 5 pA.

Remarkably, the scanning by the STM tip seemed to play a role in the growth of the edge-on domains of **MnCu2**. In the STM image in Figure 5A an area with edge-on oriented assemblies of **MnCu2** is shown, directly after scanning it after the application of a voltage pulse. The next STM image (Figure 5B) is a zoom-out in which the scanning area was enlarged from 150 × 150 to 300 × 300 nm^2^. The square drawn in this image corresponds with the scanning area of Figure 5A. It can be clearly seen that the edge-on assemblies are predominantly present in the confined area of the previous scan. The STM image in Figure 5C shows the same area as in Figure 5B, but now after 20 min of continuous scanning. A large part of the scanning area is now covered with the ‘edge-on’ assemblies, which indeed indicates that the STM tip plays an active role in their growth and that the domains are formed from a seed of ‘edge-on’ oriented molecules, as described above. The precise mechanism by which the tip assists in the domain growth is as yet unknown. With the applied tunneling parameters (V_bias_ = −450 mV, I_t_ = 5 pA) the tip is relatively far removed from the surface, minimizing direct physical contact with the monolayer. The porphyrins are not very polar, but to some extent polarizable along their planar axes. The electric field in the tip-sample junction is rather high (in the regime of 10^9^ V/m). Polar and polarizable species may form bridges in electric fields, which can act as nuclei for further growth with a molecular orientation of the most polarizable direction along the field direction, i.e., in this case, perpendicular to the surface. This effect is best known for water molecules [54], which are polar, however, a static polarization during the pulse duration may suffice to initiate growth nuclei of polarizable species of such an ‘upright’ configuration. Furthermore, the movement of the tip may perturb the supernatant solution containing molecules or assemblies of **MnCu2** and thereby provide them with energy to self-assemble into ordered arrays at the solid–liquid interface.

## 3. Materials and Methods

### 3.1. General Materials and Methods

All commercially obtained chemicals were used without further purification unless stated otherwise. CHCl_3_ (Merck, Burlington, MA, USA) was distilled under nitrogen from calcium chloride. Toluene (Merck, Burlington, MA, USA) was distilled under nitrogen from sodium. Pyrrole (Sigma-Aldrich Co. St. Louis, MO, USA) was freshly distilled before use. For TLC analysis, TLC Silicagel 60 F254 (Merck, Burlington, MA, USA) and for column chromatography Silica gel 0.035–0.070 mm, 60A (Acros, Branchburg, N.J., USA) or Alumina 0.050–0.200 mm 60A activity I (Acros) were used. ^1^H and ^13^C-NMR spectra were recorded on Varian Inova 400 MHz or Bruker Avance 500 MHz spectrometers at 25 °C. Chemical shifts are reported in parts per million (ppm) relative to tetramethylsilane (TMS, 0.00 ppm) as the internal reference. Multiplicities used are: s = singlet, bs = broad singlet, d = doublet, dd = doublet of doublets, bd = broad doublet, t = triplet, q = quartet, m = multiplet and/or multiple overlapping resonances). Phase and baseline correction was applied to all NMR spectra (Bruker, Billerica, MA, USA). MALDI-TOF mass spectra were measured in reflective mode with dithranol as a matrix on a Bruker Microflex LRF MALDI-TOF mass spectrometer (Bruker, Billerica, MA, USA). All chemical reactions were carried out under an argon atmosphere. Computational studies were performed by using the molecular modeling program Spartan14 and the energy minimization calculations were performed using the semi-empirical PM3 method.

### 3.2. Syntheses

**4-Octadecyloxy-benzaldehyde** (**3**)**.** A mixture of 1-bromooctadecane (24.5 g, 73.4 mmol, 4-hydroxybenzaldehyde (8.15 g, 66.7 mmol, and K_2_CO_3_ (27.7 g, 200 mmol) in DMF (100 mL) was stirred under argon at 60 °C for 4 h. After cooling, the solvent was evaporated and the residue dissolved in CHCl_3_ (150 mL). This solution was washed with aqueous 0.1 N HCl (2×) and water and evaporated to dryness. The crude product was recrystallized from methanol to yield 4-octadecyloxy-benzaldehyde as a white solid (19.5 g, 78%). ^1^H-NMR (400 MHz, CDCl_3_) δ 9.88 (s, 1H), 7.83 (d, 2H, J = 8.8 Hz), 6.99 (d, 2H, J = 8.8 Hz), 4.40 (t, 2H, J = 6.8 Hz), 1.85–1.77 (m, 2H), 1.49–1.41 (m, 2H), 1.41–1.19 (m, 30H), 0.88 (t, 3H, J = 7.0 Hz) ppm. ^13^C-NMR (100 MHz, CDCl_3_) δ 190.78, 164.25, 131.97, 129.73, 114.73, 68.43, 31.92, 29.68, 29.35, 29.05, 25.95, 22.69, 14.11 ppm. 

**5,10,15,20-Tetrakis-(4-octadecyloxy-phenyl)-porphyrin** (**4**)**.** Compound **3** (5.107 g, 13.63 mmol) and pyrrole (915 mg, 13.63 mmol) were dissolved in propionic acid (100 mL). The mixtures were stirred at reflux for 4.5 h) After cooling, the solvent was evaporated and the crude product was purified by column chromatography (alumina, eluent: CHCl_3_). The resulting product was recrystallized 3 times from methanol to yield **4** as a purple solid (2.17 g, 38%). ^1^H-NMR (500 MHz, CDCl_3_) δ 8.86 (s, 8H), 8.10 (d, 8H, J = 9.0 Hz), 7.27 (d, 8H, J = 9.0 Hz), 4.25 (t, 8H, J = 6.5 Hz), 2.03–1.93 (m, 8H), 1.67–1.57 (m, 8H), 1.55–1.19 (m, 112H), 0.87 (t, 12H, J = 7.0 Hz), −2.75 (bs, 2H) ppm. ^13^C-NMR (125 MHz, CDCl_3_) δ 158.95, 135.58, 134.45, 119.79, 112.69, 68.36, 31.93, 29.72, 29.69, 29.66, 29.55, 29.36, 26.26, 22.69, 14.12 ppm. MALDI-TOF calculated for C_116_H_174_N_4_O_4_: m/z 1688.7; measured: 1688.1 (M)^+^. UV-vis (CHCl_3_) λ/nm 423, 519, 557, 593, 650.

**5,10,15,20-Tetrakis-(4-octadecoxy-phenyl)-2,3,21,23-tetrahydro-porphine-2,3-diol** (**5**)**.** To a stirred solution of **4** (2.39 g, 1.41 mmol) in a mixture of CHCl_3_ (180 mL) and pyridine (20 mL), osmium tetroxide solution (1.11 mL, 3.54 mmol) was added, and the mixture was stirred in the dark for 5 days at room temperature. The reaction was quenched by adding a solution of sodium bisulfite (1.62 g, 15.6 mmol) in water (800 mL) and the resulting mixture was stirred for an additional 1.5 h. The organic layer was separated and washed with 1N aqueous HCl, with water, with a saturated aqueous NaHCO_3_ solution, dried over MgSO_4_, and evaporated to dryness. The product was purified by column chromatography (Silica, eluent: gradient, CHCl_3_ to 3% MeOH in CHCl_3_ (v/v). Compound **5** was obtained as a red/purple solid (1.0 g, 0.58 mmol, 41%). ^1^H-NMR (400 MHz, CDCl_3_) δ 8.62 (bd, 4H, J = 4.8 Hz), 8.58 (d, 2H, J = 4.8 Hz), 8.46 (s, 2H), 8.29 (d, 2H, J = 4.8 Hz), 7.99 (bd, 4H, J = 6.4 Hz), 7.94 (bd, 1H, J = 7.7 Hz), 7.86 (bd, 1H, J = 6.8 Hz), 7.82–7.75 (m, 2H), 7.41–7.33 (m, 4H), 7.20 (d, 4H, J = 8.9 Hz), 7.14–7.07 (m, 1H), 6.99 (s, 2H), 6.91–6.85 (m, 1H), 4.21 (t, 4H, J = 6.5 Hz), 4.13 (t, 4H, J = 6.7 Hz), 2.00–1.87 (m, 8H), 1.65–1.20 (m, 120H), 0.88 (t, 12H, J = 6.7 Hz), −1.73 (bs, 2H) ppm. MALDI-TOF calculated for C_116_H_176_N_4_O_6_: m/z 1722.7; measured: 1723.2 (M + H)^+^, 1704.2 (M – OH + H)^+^, 1689.2 (M − 2OH + H)^+^. UV-vis (CHCl_3_) λ/nm (log(ε/M^−1^cm^−1^) 424 (5.02), 525 (3.93), 555 (3.98), 596 (3.73), 648 (3.98). 

**5,10,15,20-Tetrakis-(4-octadecoxy-phenyl)-porphyrin-2,3-dione** (**6**). Compound **5** (1.0 g, 0.58 mmol) was dissolved in distilled toluene and dry pyridine. 4,5-Dichloro-3,6-dioxocyclohexa-1,4-diene-1,2-dicarbonitrile (DDQ) (0.546 g, 2.41 mmol) was added and the mixture was stirred at reflux for 24 h. After cooling, the mixture was evaporated to dryness and taken up in dichloromethane, which was filtered through celite. The resulting filtrate was evaporated to dryness and the product was purified by column chromatography (Silica, eluent: CH_2_Cl_2_: n-heptane 1:1, v/v), yielding **6** as a brown/yellow solid (0.38 g, 38%). ^1^H NMR (400 MHz, CDCl_3_) δ 8.76 (dd, 2H, J = 5.0 Hz, J = 1.0 Hz), 8.62 (dd, 2H, J = 5.0 Hz, J = 1.0 Hz), 8.58 (s, 2H), 8.03–7.98 (m, 4H), 7.80–7.74 (m, 4H), 7.27–7.22 (m, 4H), 7.22–7.16 (m, 4H), 4.25–4.16 (m, 8H), 2.01–1.89 (m, 8H), 1.66–1.55 (m, 8H), 1.52–1.18 (m, 112H), 0.87 (s, 12H, J = 6.8 Hz), −1.96 (bs, 2H) ppm. ^13^C NMR (125 MHz, CDCl_3_) δ 188.18, 159.35, 159.18, 155.74, 140.83, 140.20, 138.34, 135.34, 134.25, 133.79, 133.36, 131.42, 128.42, 128.07, 124.01, 113.53, 113.23, 113.02, 68.39, 68.20, 31.93, 29.73, 29.68, 29.55, 29.48, 29.37, 26.25, 22.70, 14.12 ppm. MALDI-TOF calculated for C_116_H_172_N_4_O_6_: m/z 1718.6; measured: 1719.4 (M + H)^+^. UV-vis (CHCl_3_) λ/nm (log(ε/M^−1^cm^−1^) 410 (5.26), 479 (4.28).

**Porphyrin dimer H_4_1**. Compound **6** (105 mg, 0.061 mmol) and benzene-1,2,4,5-tetraamine tetrahydrochloride (8.25 mg, 0.029 mmol) were dissolved in pyridine (35 mL) and the solution was refluxed with solvent running back through a soxhlet condenser containing mol sieves (4 Å). After 60 h, the mixture was allowed to cool and subsequently evaporated to dryness. The product was purified by column chromatography (Silica, eluent: CH_2_Cl_2_:n-heptane 1:1, v/v), followed by purification by size-exclusion chromatography (BioBeads, eluent: toluene). The first fraction contained the desired product, which was redissolved in a minimal amount of CHCl_3_. This solution was added to an excess of stirred methanol, which caused the product to precipitate. After filtration, **H_4_1** was obtained as a red/brown solid (13.4 mg, 13%). ^1^H NMR (500 MHz, CDCl_3_) δ 9.00 (d, 2H, J = 4.9 Hz), 8.94 (d, 2H, J = 4.9 Hz), 8.77 (s, 2H), 8.74 (s, 4H), 8.20–8.12 (m, 16H), 7.41 (d, 8H, J = 8.5 Hz), 7.31 (d, 8H, J = 8.6 Hz), 4.50 (t, 8H, J = 6.2 Hz), 4.27 (t, 8H, J = 6.4 Hz), 2.32–2.23 (m, 8H), 2.05–1.96 (m, 8H), 1.88–1.78 (m, 8H), 1.69–1.57 (m, 16H), 1.54–1.46 (m, 8H), 1.46–1.14 (m, 200H), 0.88 (t, 12H, J = 6.8 Hz), 0.84 (t, 12H, J = 6.9 Hz), −2.31 (bs, 4H) ppm. ^13^C NMR (125 MHz, CDCl_3_) δ 159.52, 159.15, 155.30, 153.35, 145.85, 140.21, 139.50, 135.63, 135.21, 134.05, 133.94, 127.96, 121.74, 116.35, 113.06, 112.88, 68.54, 68.38, 31.93, 29.94, 29.73, 29.68, 29.55, 29.37, 29.33, 26.56, 26.27, 22.70, 14.13 ppm. MALDI-TOF calculated for C_238_H_346_N_12_O_8_: m/z 3503.4; measured: 3504.1 (M + H)^+^. UV-vis (CHCl_3_) λ/nm (log(ε/M^−1^cm^−1^) 431 (5.61), 462 (5.56), 631 (4.42).

**Copper porphyrin dimer Cu_2_1.** Porphyrin dimer **H_4_1** (2.3 mg, 0.66 µmol) was dissolved in a mixture of CHCl_3_ (20 mL) and MeOH (5 mL). Copper acetate monohydrate (1.33 mg, 6.66 mmol) was added and the mixture was heated at reflux for 15 h. After cooling, the solvent was evaporated and the product was purified by column chromatography (Silica, eluent: CHCl_3_/n-heptane 1:2, v/v, R_f_ = 0.025) to yield **Cu_2_1** as an orange/red solid (2.3 mg, 97%). MALDI-TOF calculated for C_238_H_342_N_12_O_8_Cu_2_: m/z 3626.4; measured: 3627.4 (M + H)^+^. UV-vis (CHCl_3_) λ/nm (log(ε/M^−1^cm^−1^) 428 (5.50), 457 (5.39), 517 (4.88), 543 (4.84), 675 (4.38).

**Manganese porphyrin dimer Mn_2_1.** Porphyrin dimer **H_4_1** (3.0 mg, 0.9 µmol) was dissolved in a mixture of CHCl_3_ (3 mL) and MeOH (3 mL). Manganese diacetate tetrahydrate (2.1 mg, 8.6 µmol) was added and the mixture was heated at reflux for 16 h. After cooling, brine (10 mL) was added and the mixture was vigorously stirred for 8 h. The layers were separated and the organic layer was washed with water, dried over MgSO_4_ and evaporated to dryness. The product was purified by column chromatography (Silica, eluent: 5% MeOH in CHCl_3_, v/v, R_f_ = 0.55) to yield **Mn_2_1** as a green/brown solid (3.1 mg, 98%). MALDI-TOF calculated for C_238_H_342_Cl_2_N_12_O_8_Mn_2_: m/z 3678.5; measured: 3610.2 (M − 2Cl + H)^+^. UV-vis (CHCl_3_) λ/nm (log(ε/M^−1^cm^−1^) 432 (5.50), 500 (5.32), 655 (4.69).

**Manganese porphyrin dimer Mn_2_2.** Porphyrin dimer **H_4_2** (3.0 mg, 0.9 µmol) was dissolved in a mixture of CHCl_3_ (3 mL) and MeOH (3 mL). Manganese diacetate tetrahydrate (2.1 mg, 8.6 µmol) was added and the mixture was heated at reflux for 16 h. After cooling, brine (10 mL) was added and the mixture was vigorously stirred for 8 h. The layers were separated and the organic layer was washed with water, dried over MgSO_4_ and evaporated to dryness. The product was purified by column chromatography (Silica, eluent: 5% MeOH in CHCl_3_, v/v, R_f_ = 0.7) to yield **Mn_2_1** as a dark green solid (3.1 mg, 98%). MALDI-TOF calculated for C_198_H_270_Cl_2_N_8_O_6_Mn_2_: m/z 3037.9; measured: 2967.7 (M − 2Cl)^+^, 3002.5 (M − Cl)^+^. UV-vis (CHCl_3_) λ/nm (log(ε/M^−1^cm^−1^) 339 (4.85), 382 (5.02), 406 (5.00), 482 (5.34), 584 (4.28), 625 (4.46).

### 3.3. STM Experiments

Approximately 10 μL droplet of a solution of a porphyrin dimer (concentration ~10^−4^ M) was applied to a freshly cleaved HOPG substrate (10 × 10 mm^2^, NT-MDT, ZYB) which was mounted into a home-built liquid-STM setup [25]. The STM-tip (mechanically cut from Pt_0.8_Ir_0.2_ wire, diameter 0.5 mm) was immersed in the droplet and the surface was scanned at a rate of 300 nm/s. To minimize thermal drift effects, the STM setup was mounted and left to equilibrate for several hours before the solution containing the compound was added. The STM measurements were performed in constant-current mode using an Omicron Scala SPM controller. The images shown were only corrected for background and no additional filters were applied to the raw data. All STM measurements were performed in the thermostatted environment (21.5 ± 0.5 °C) of the NanoLab Nijmegen.

## 4. Conclusions

A porphyrin dimer with a rigid aromatic linker was readily synthesized in five steps, and its bis-copper and bis-manganese complexes were obtained nearly quantitatively from the free base precursor. The bis-copper dimer self-assembled into stable monolayers at a solid–liquid interface. The bis-manganese dimer formed monolayers with a similar structure, but these were much less stable than the layers of the bis-copper dimers. Similar unstable layers were observed for bis-manganese porphyrin dimers in which the porphyrins are linked by a less-rigid alkyne spacer. The instability of both layers is attributed to the presence of inner shell axial chloride ligands at the manganese centers, which are believed to hinder a cofacial orientation of the porphyrin planes with respect to the surface.

The application of a mild voltage pulse induced the emergence of an adlayer of a copper-manganese porphyrin dimer with an alkyne spacer, in which the dimer molecules were exclusively oriented with their porphyrin planes perpendicular to the surface. The formation of this surface polymorph was attributed to (i) dissociation of the axial chloride ligand from the manganese center allowing cofacial intermolecular stacking, and/or (ii) electric field-induced preorganization of small stacks in solution which, after adsorption to the surface, serve as seeds for further growth of the edge-on polymorph.

Future research will be directed to investigating the (cooperative) reactivity of the bis-manganese dimers towards molecular oxygen. In addition, these dimers will be equipped with non-coordinating anions (e.g., PF_6_^−^ or BPh_4_^−^) via ion-exchange reactions, to improve their adsorption at a solid–liquid interface into more stable adlayers.

## Supplementary Materials

The following are available online: ^1^H and ^13^C-NMR, mass, and UV-vis spectra of all new compounds.
