# Supplementary material for: Self-Assembly of Covalently Linked Porphyrin Dimers at the Solid–Liquid Interface
Source: Molecules. 2019 Aug 20;24(16):3018. doi: 10.3390/molecules24163018 (PMC6720350; doi:10.3390/molecules24163018)
Supplement: Supplementary file 1 [file molecules-24-03018-s001.pdf]

Supporting Information

Self-Assembly of Covalently Linked Porphyrin Dimers at the Solid-Liquid Interface

Thomas Habets[1,#], Dennis Lensen[1,#], Sylvia Speller[2] and Johannes A. A. W. Elemans[1,*]

[1] Radboud University, Institute for Molecules and Materials, Heyendaalseweg 135, 6525 AJ Nijmegen, The Netherlands
[2] University of Rostock, Institute of Physics, Albert-Einstein-Straße 23, 18059 Rostock, Germany
[#] These authors contributed equally
* Correspondence: j.elemans@science.ru.nl.

Contents

Figure S1 ^{1}H NMR and ^{13}C NMR spectra of compound **3**. 2
Figure S2 ^{1}H NMR and ^{13}C NMR spectra of compound **4**. 3
Figure S3 MALDI-TOF spectrum of compound **4**. 4
Figure S4 UV-vis spectrum of compound **4**. 4
Figure S5 ^{1}H NMR spectrum of compound **5**. 5
Figure S6 MALDI-TOF spectrum of compound **5**. 5
Figure S7 UV-vis spectrum of compound **5**. 6
Figure S8 ^{1}H NMR and ^{13}C NMR spectra of compound **6**. 7
Figure S9 MALDI-TOF spectrum of compound **6**. 8
Figure S10 UV-vis spectrum of compound **6**. 8
Figure S11 ^{1}H NMR and ^{13}C NMR spectra of compound **$H_4$1**. 9
Figure S12 ^{1}H-^{13}C NMR correlation and ^{13}C-DEPT135 NMR spectra of compound **$H_4$1**. 10
Figure S13 MALDI-TOF spectrum of compound **$H_4$1**. 11
Figure S14 UV-vis spectrum of compound **$H_4$1**. 11
Figure S15 MALDI-TOF spectrum of compound **$Cu_2$1**. 12
Figure S16 UV-vis spectrum of compound **$Cu_2$1**. 12
Figure S17 MALDI-TOF spectrum of compound **$Mn_2$1**. 13
Figure S18 UV-vis spectrum of compound **$Mn_2$1**. 13
Figure S19 MALDI-TOF spectrum of compound **$Mn_2$2**. 14
Figure S20 UV-vis spectrum of compound **$Mn_2$2**. 14

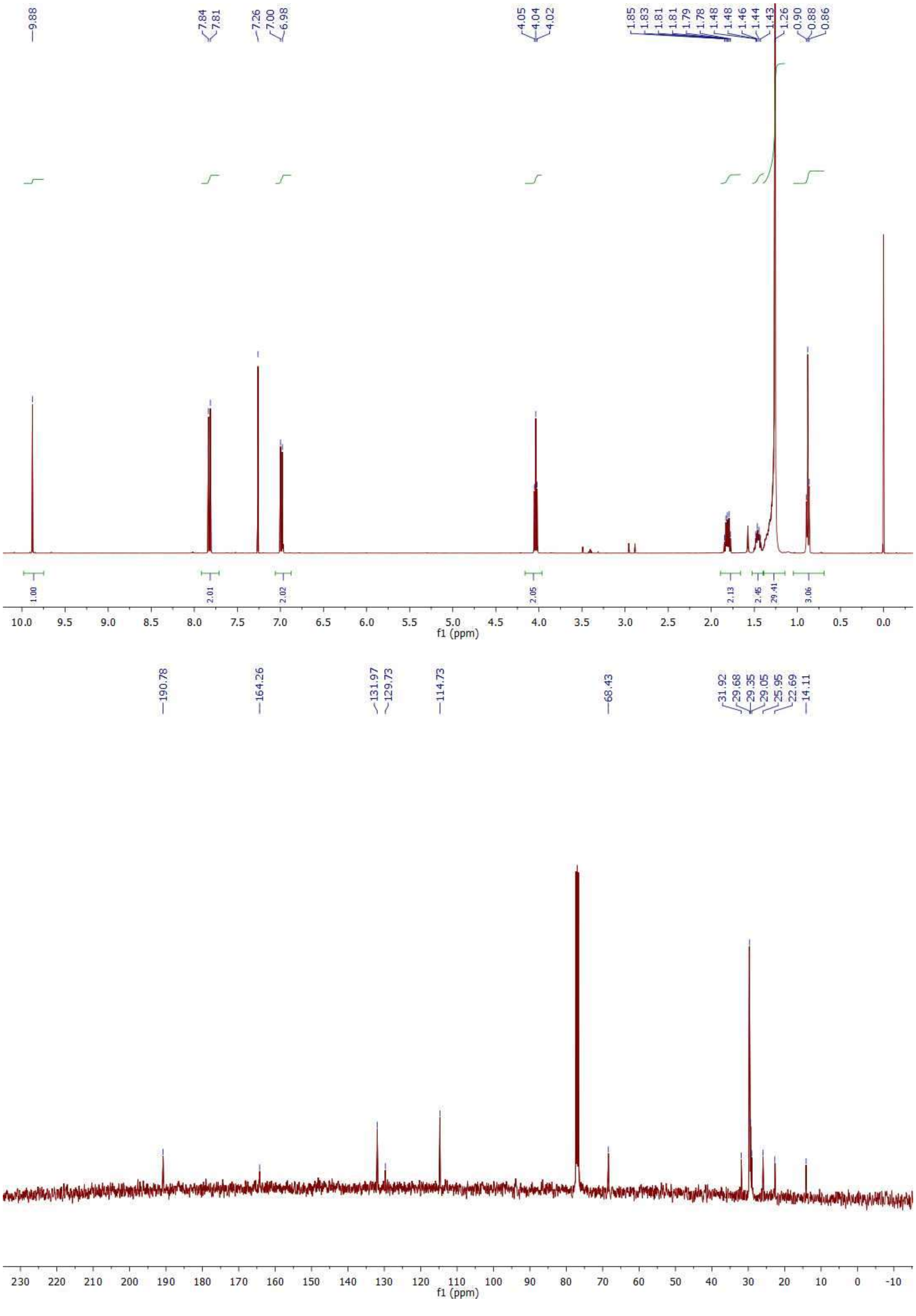

Figure S1 ^{1}H NMR (400 MHz, $CDCl_3$) (top) and ^{13}C NMR (100 MHz, $CDCl_3$) (bottom) spectra of compound **3**.

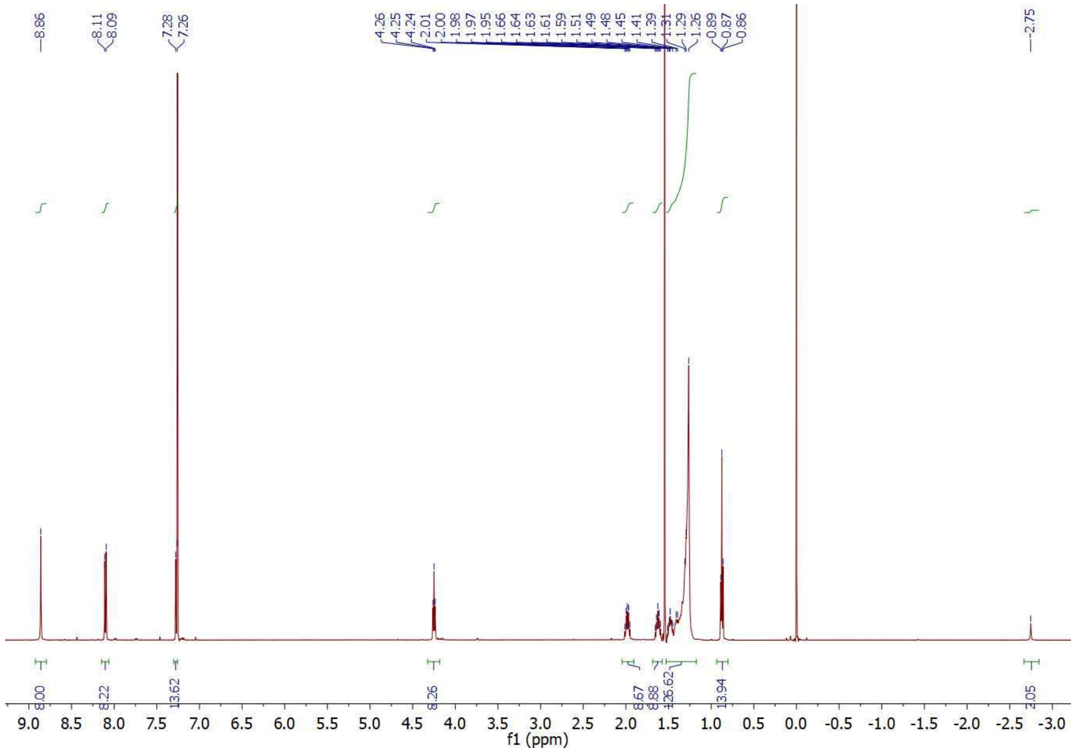

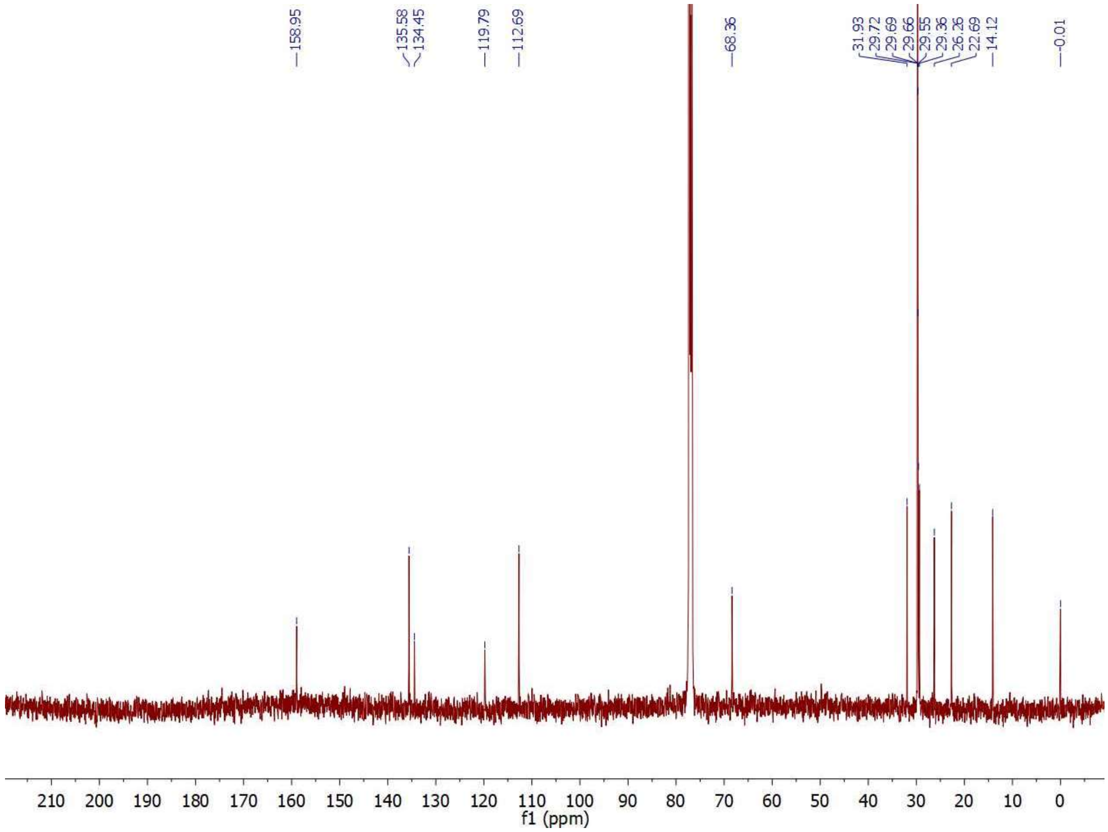

Figure S2 ^1H NMR (500 MHz, $CDCl_3$) (top) and ^{13}C NMR (100 MHz, $CDCl_3$) (bottom) spectra of compound **4**.

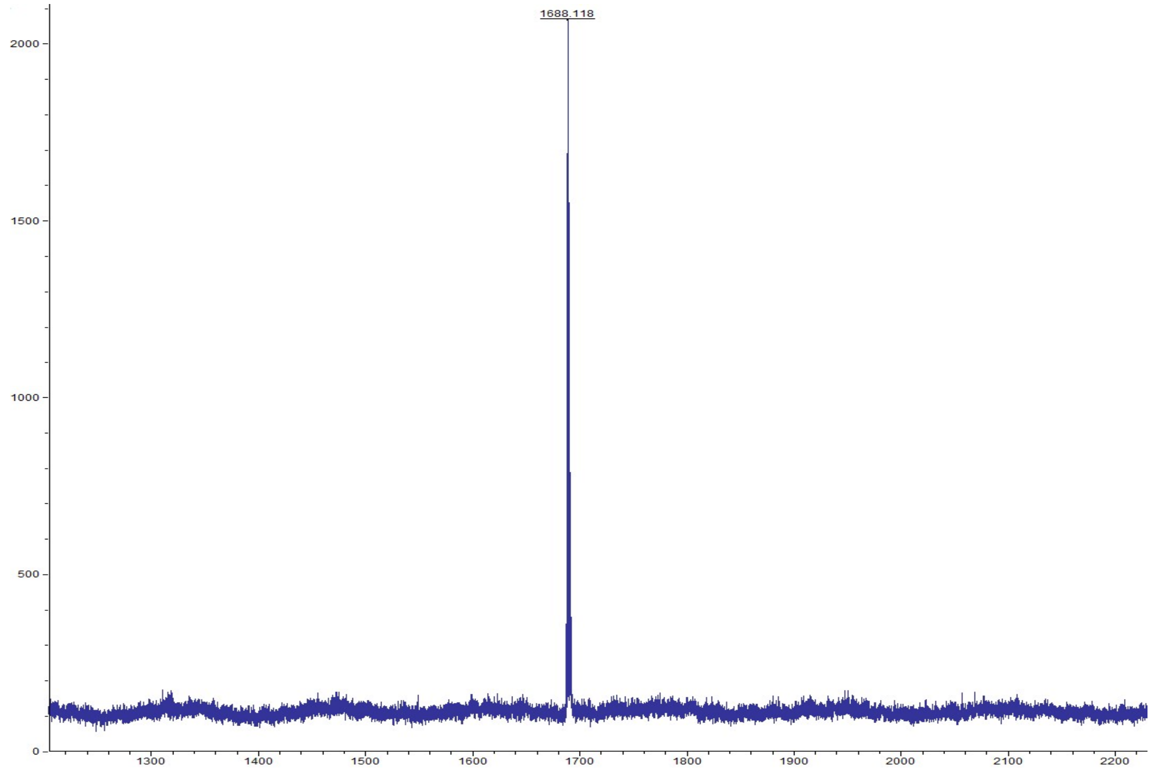

Figure S3 MALDI-TOF spectrum of compound **4**.

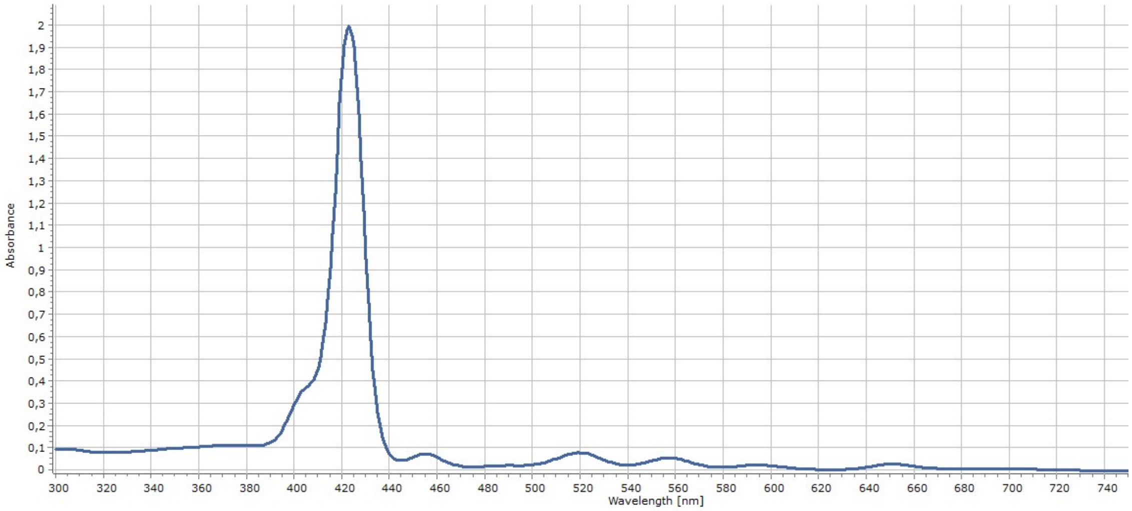

Figure S4 UV-vis spectrum of compound **4** (c = 3.2 μM) in $CHCl_3$.

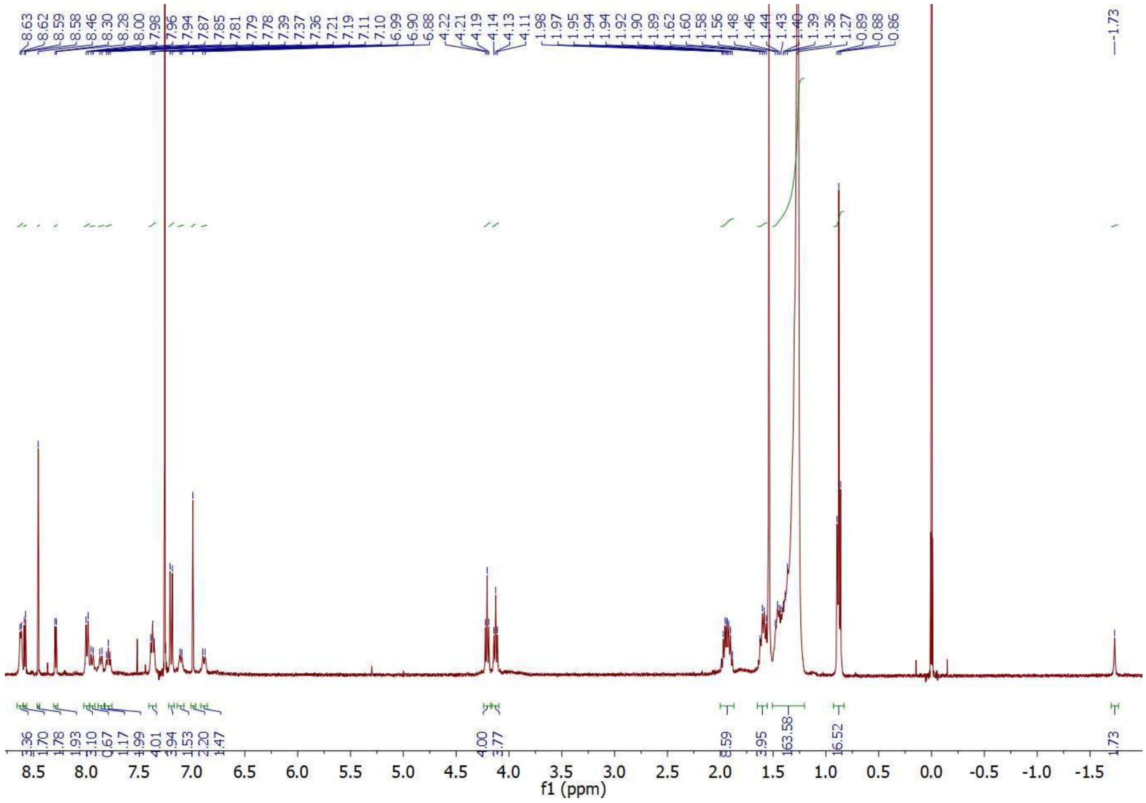

Figure S5 ^{1}H NMR (400 MHz, $CDCl_3$) spectrum of compound **5**.

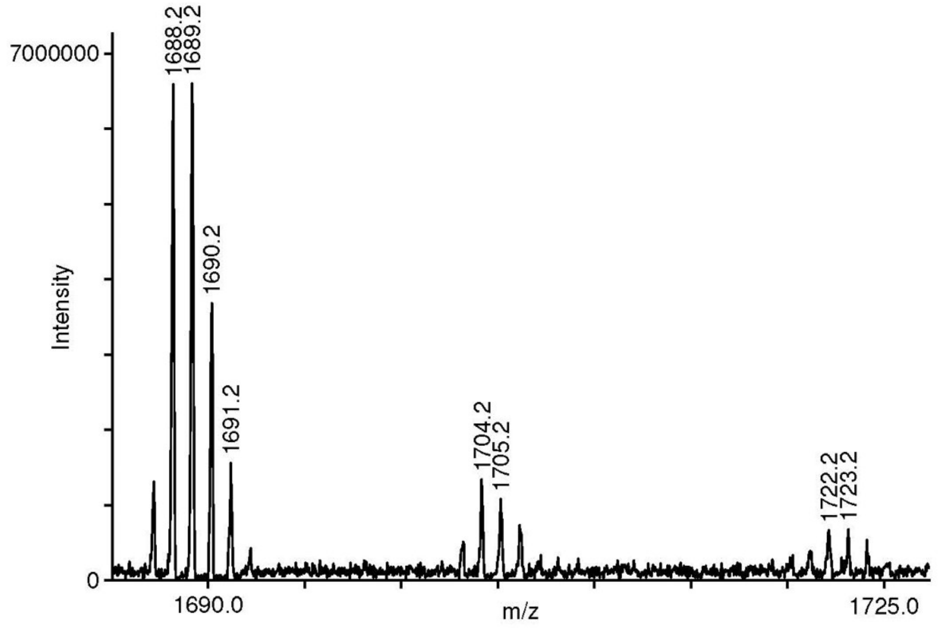

Figure S6 MALDI-TOF spectrum of compound **5**.

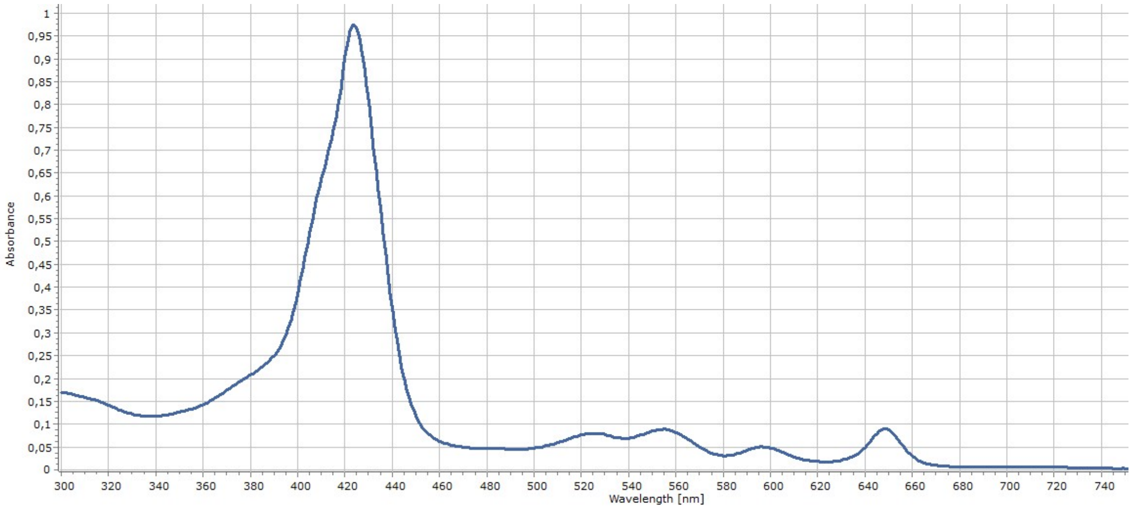

Figure S7 UV-vis spectrum of compound **5** (c = 9.3 μM) in $CHCl_3$.

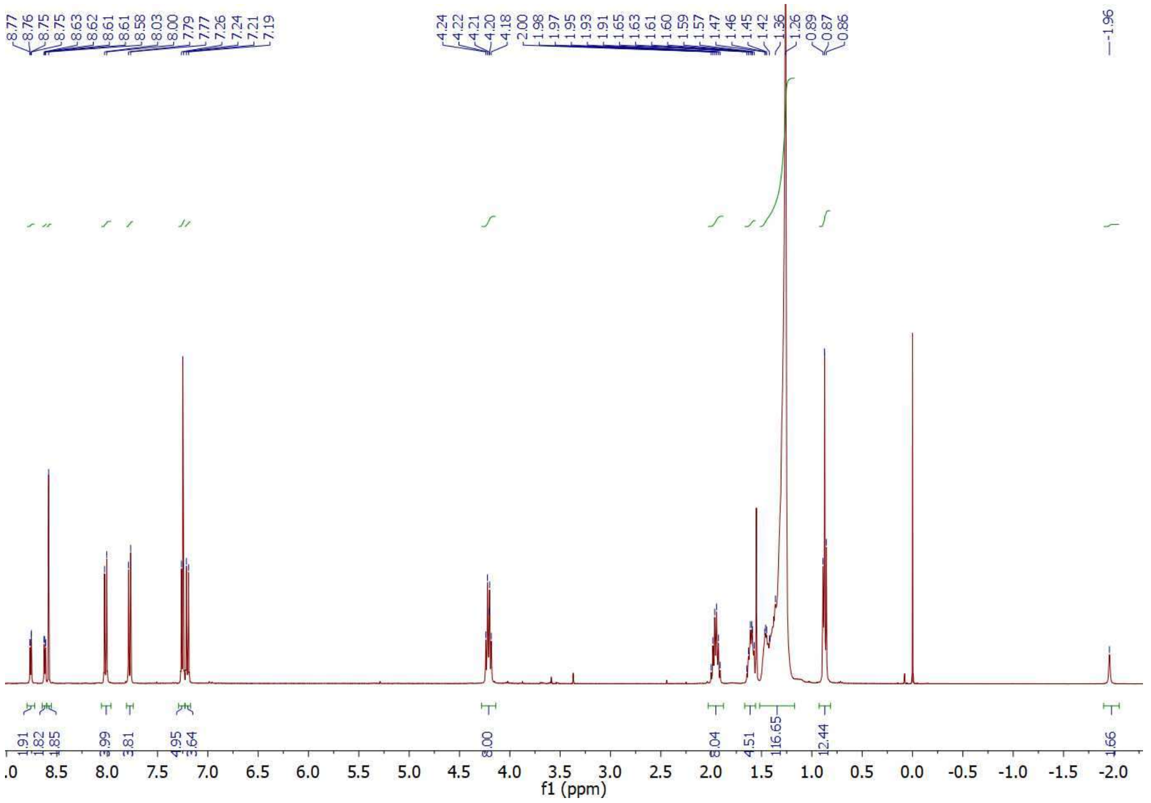

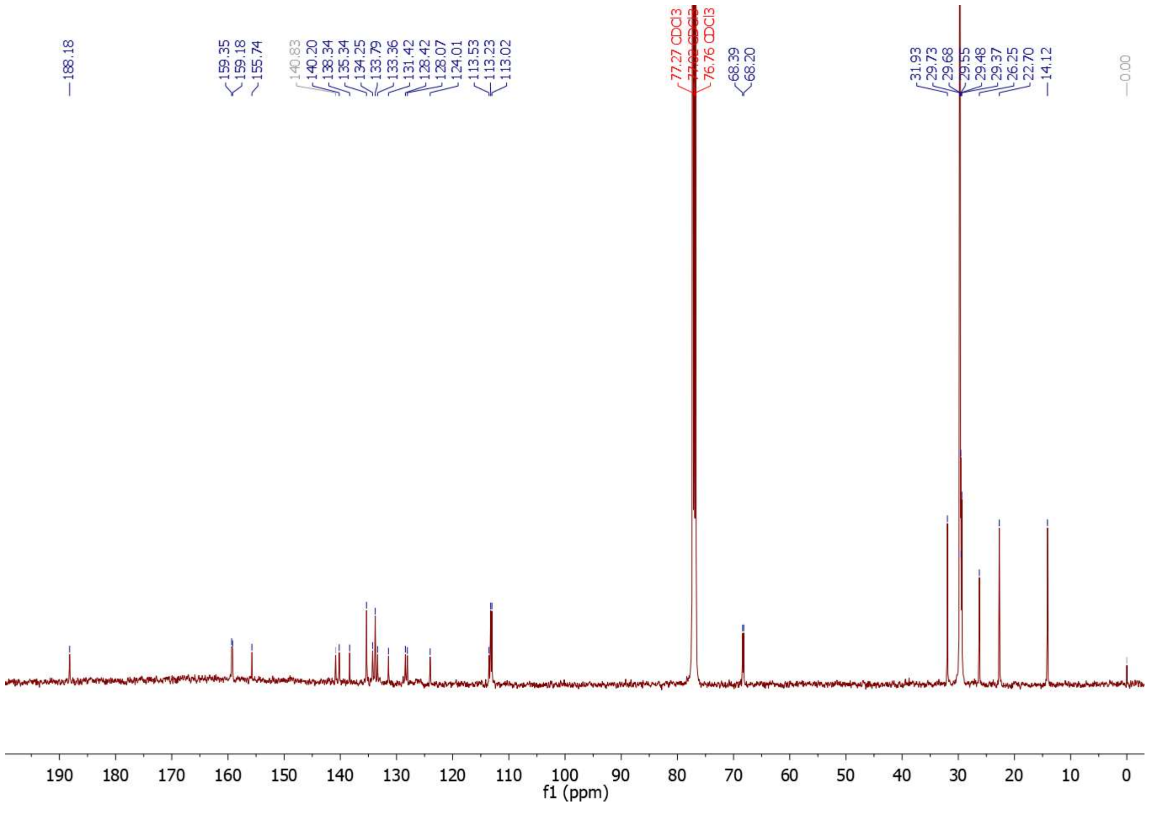

Figure S8 ^{1}H NMR (400 MHz, $CDCl_3$) (top) and ^{13}C NMR (100 MHz, $CDCl_3$) (bottom) spectra of compound **6**.

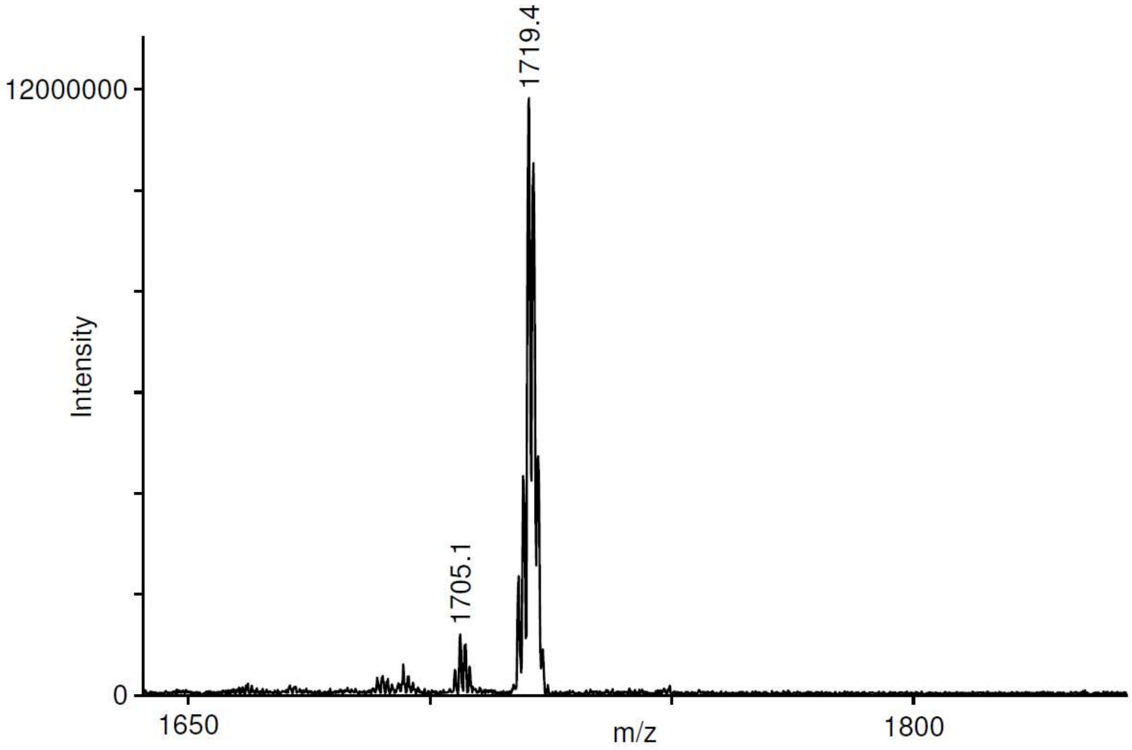

Figure S9 MALDI-TOF spectrum of compound **6**.

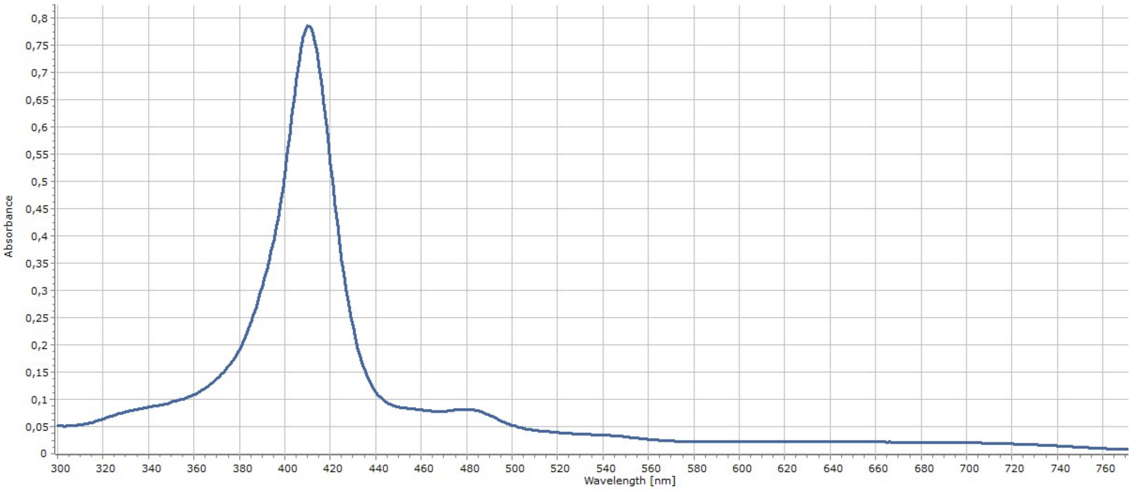

Figure S10 UV-vis spectrum of compound **6** (c = 4.31 μM) in $CHCl_3$.

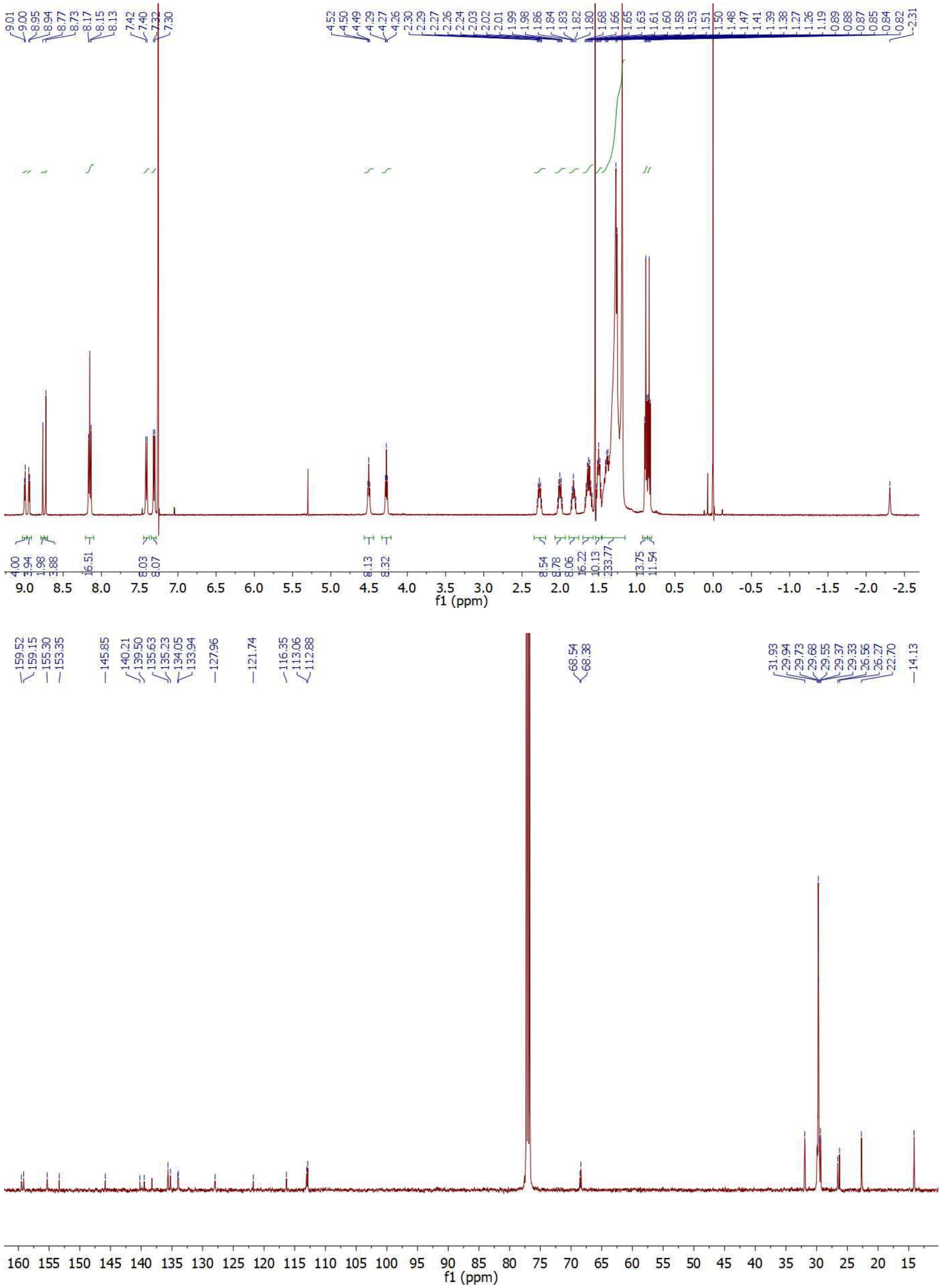

Figure S11 ^{1}H NMR (500 MHz, $CDCl_3$) (top) and ^{13}C NMR (125 MHz, $CDCl_3$) (bottom) spectrum of compound **$H_4$1**.

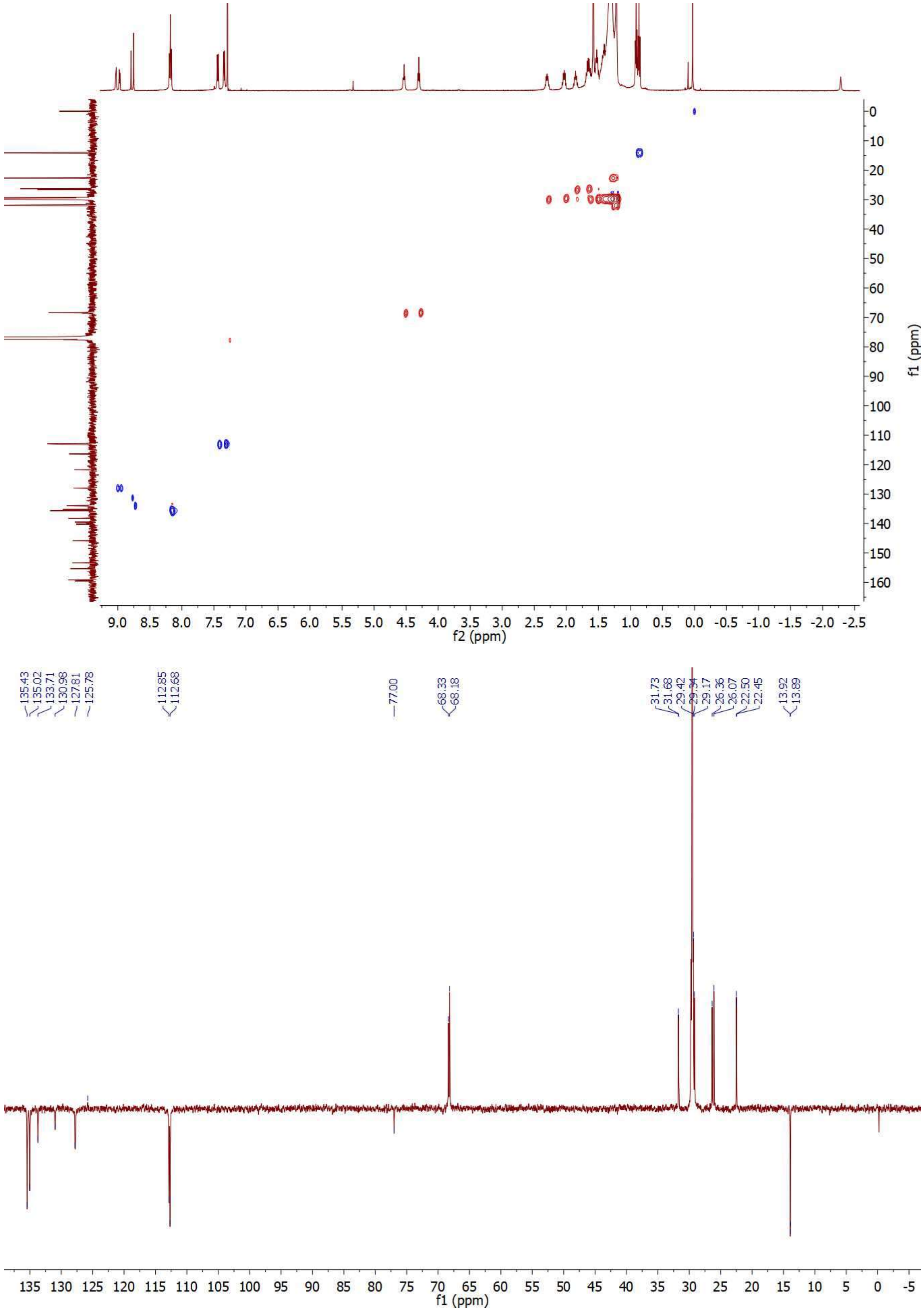

Figure S12 ^1H-^{13}C NMR correlation (500/125 MHz, $CDCl_3$) (top) and ^{13}C-DEPT135 NMR (125 MHz, $CDCl_3$) (bottom) spectra of compound $\mathbf{H_41}$.

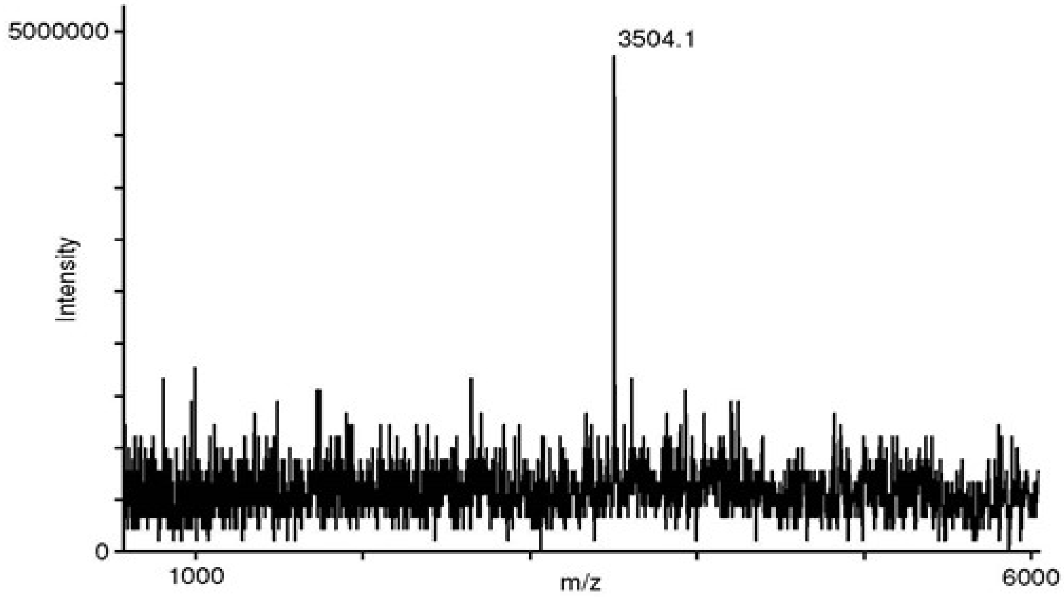

Figure S13 MALDI-TOF spectrum of compound **$H_4$1**.

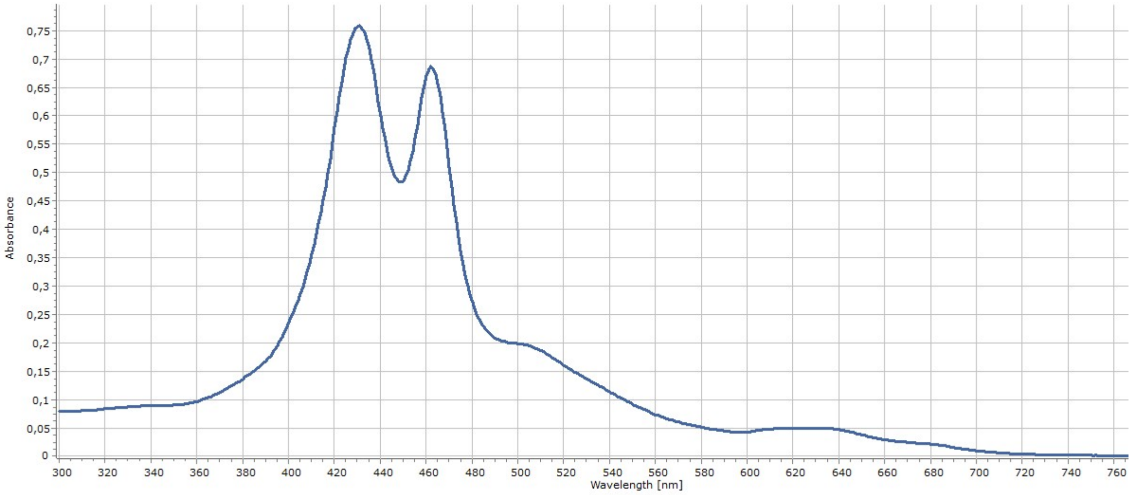

Figure S14 UV-vis spectrum of compound **$H_4$1** (c = 1.88 μM) in $CHCl_3$.

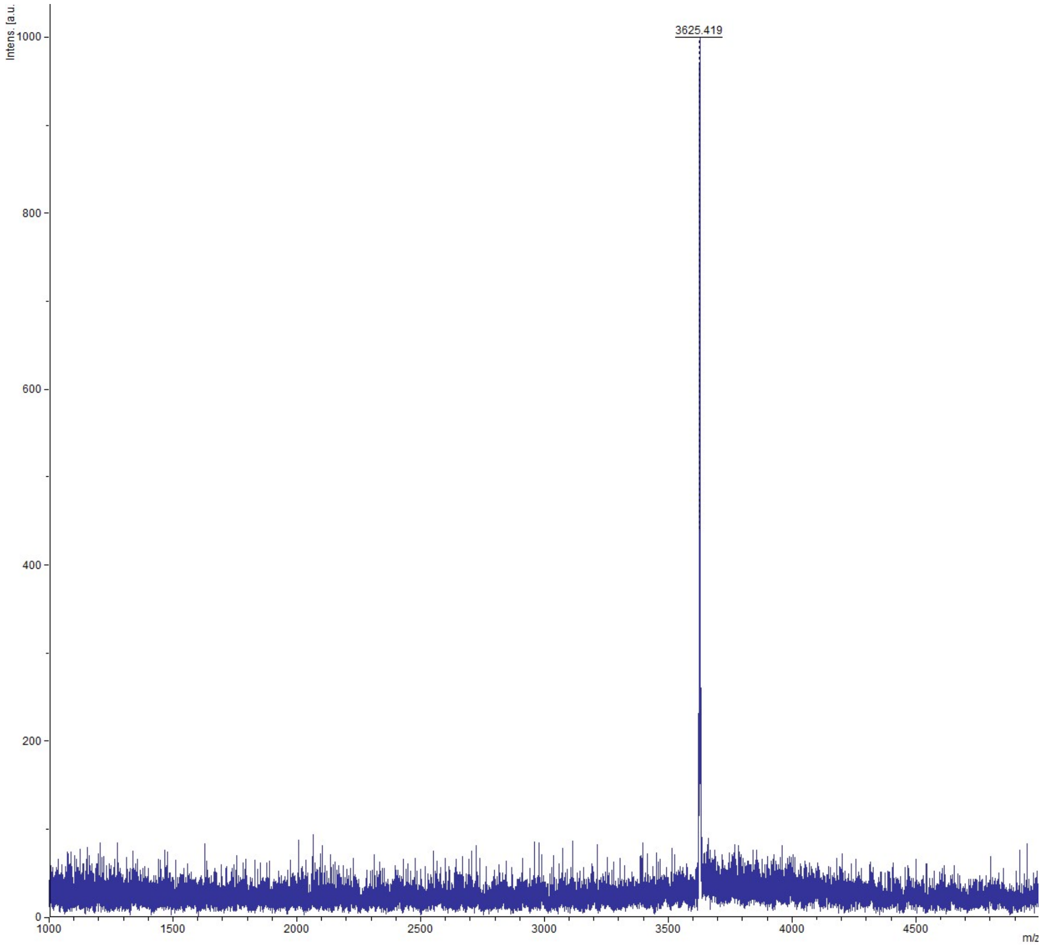

Figure S15 MALDI-TOF spectrum of compound **$Cu_2$1**.

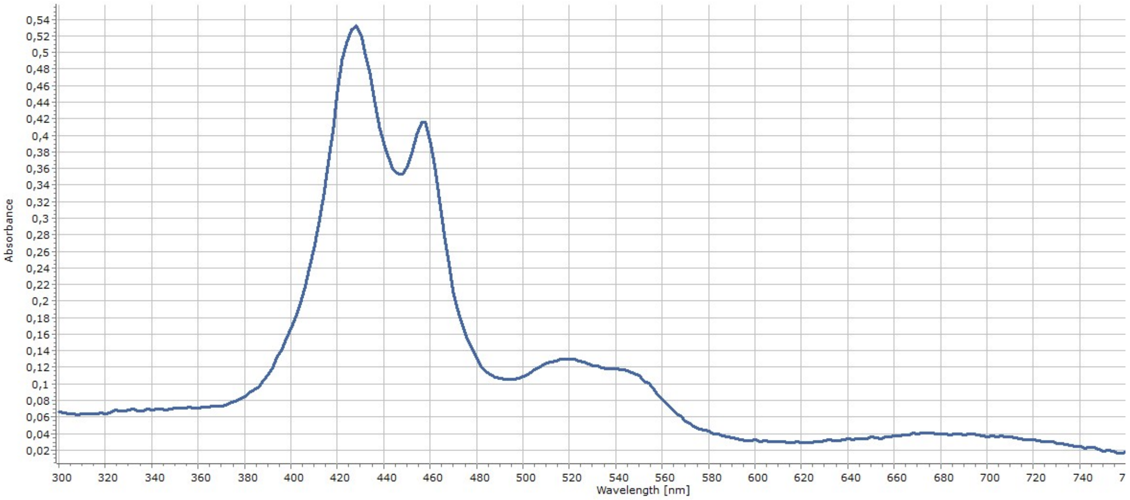

Figure S16 UV-vis spectrum of compound **$Cu_2$1** (c = 1.7 μM) in $CHCl_3$.

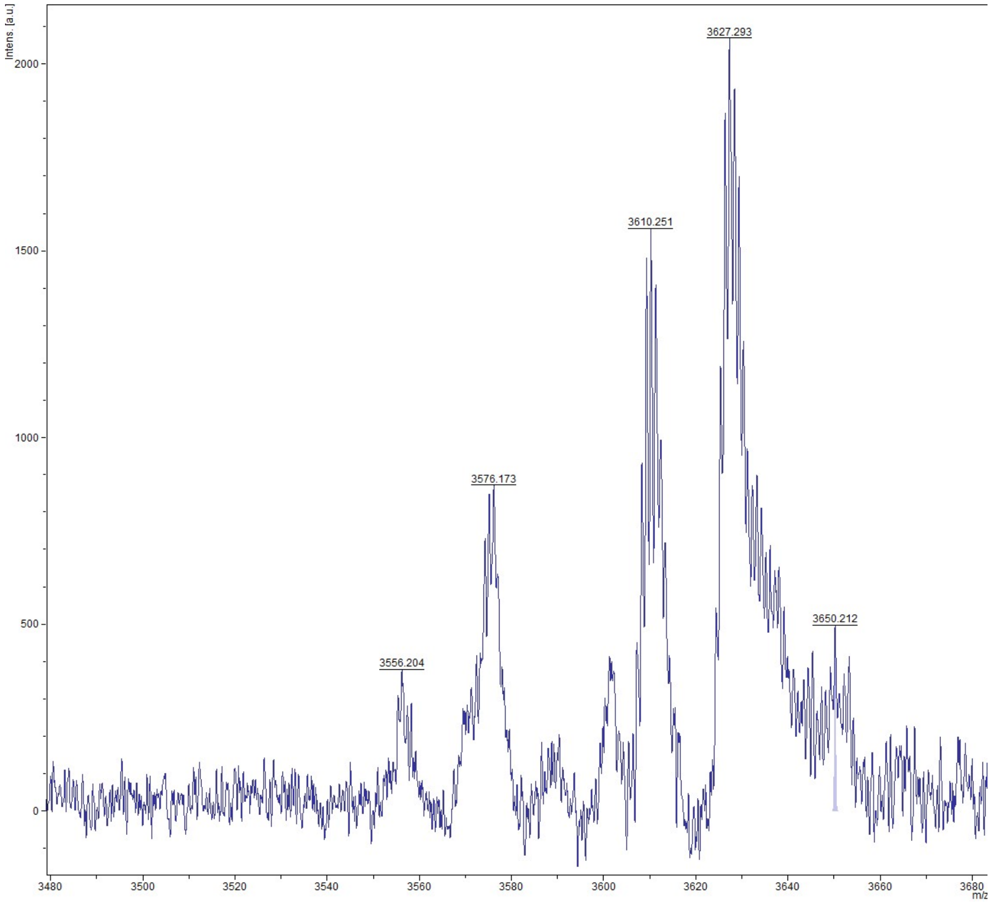

Figure S17 MALDI-TOF spectrum of compound **Mn_21**.

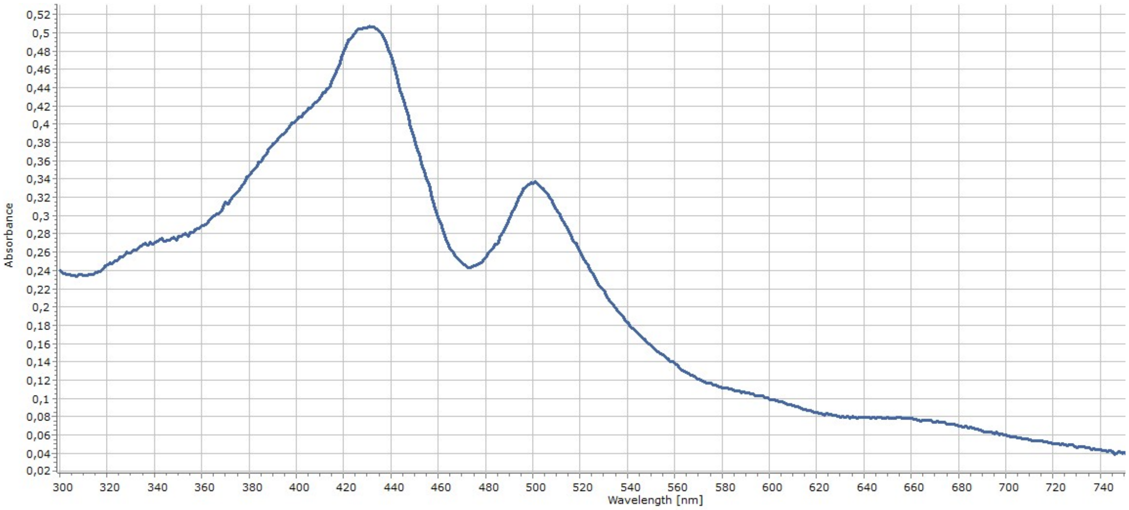

Figure S18 UV-vis spectrum of compound **Mn_21** (c = 1.6 μM) in $CHCl_3$.

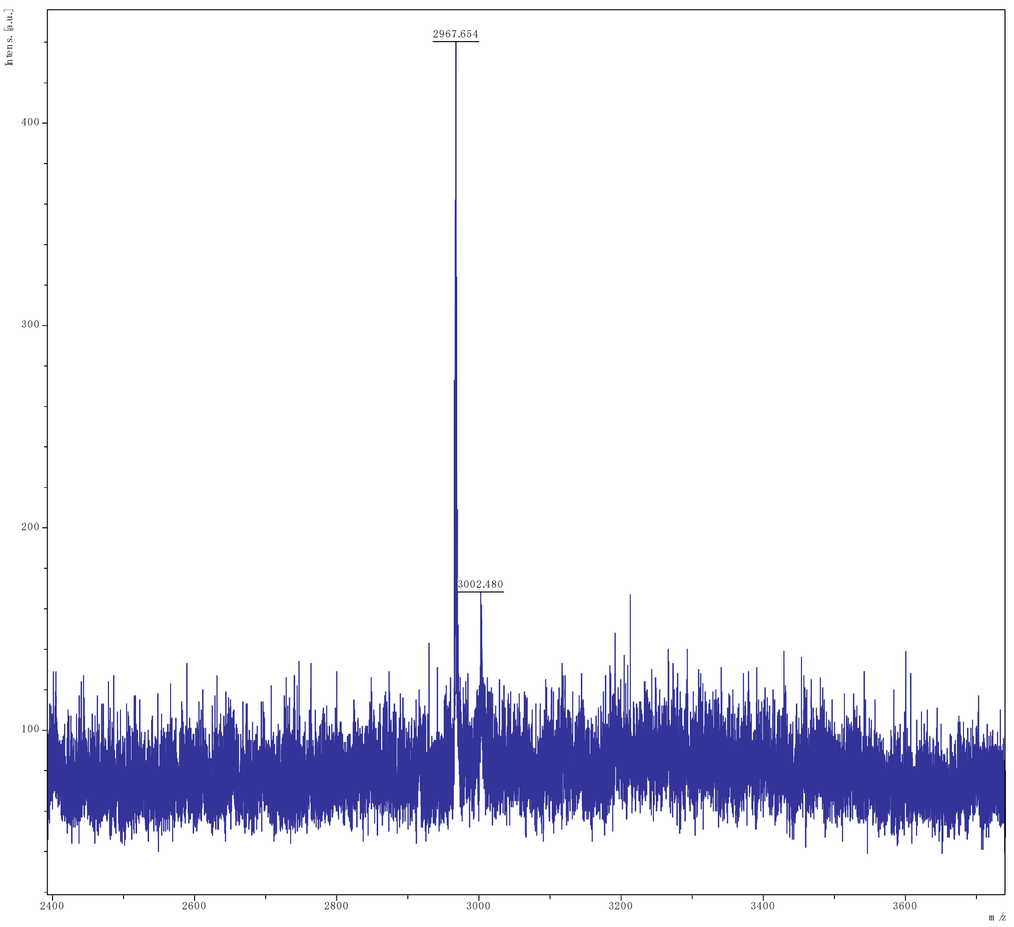

Figure S19 MALDI-TOF spectrum of compound **$Mn_2$2**.

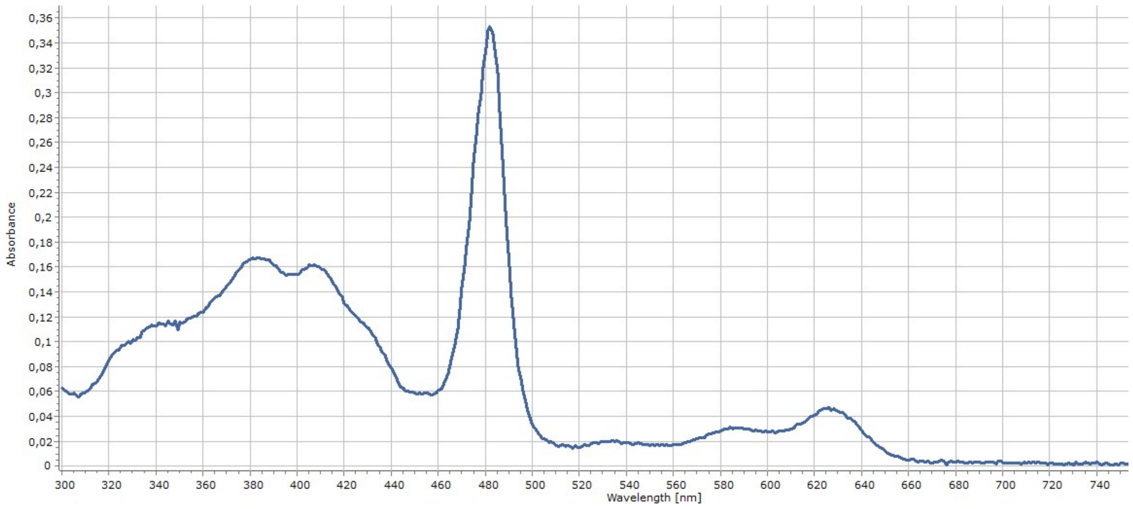

Figure S20 UV-vis spectrum of compound **$Mn_2$2** (c = 1.6 μM) in $CHCl_3$.
